# Supplementary material for: Socioeconomic position and eye health outcomes: identifying inequality in rapid population-based surveys
Source: BMJ Open. 2023 Mar 7;13(3):e069325. doi: 10.1136/bmjopen-2022-069325 (PMC10008479; doi:10.1136/bmjopen-2022-069325)
Supplement: Supplementary data [file bmjopen-2022-069325supp001.pdf]

BMJ Publishing Group Limited (BMJ) disclaims all liability and responsibility arising from any reliance placed on this supplemental material which has been supplied by the author(s)

Supplementary Material

Table of Contents

1. EquityTool questionnaire for The Gambia 1
2. Tables of results used in Figures 1-3 2

1. EquityTool questionnaire for The Gambia

Released 17 Dec 2018.

Asset list

Does your household have a… (Y/N)

1. Sofa
2. Wardrobe
3. Television
4. Refrigerator
5. DVD/CD Player
6. Satellite Dish
7. Bicycle (belonging to any member of the household)

Household Characteristics

What is the main source of drinking water for members of your household? Specify if other

1. Public tap/ Standpipe
2. Piped to yard/ plot
3. Other water source

What kind of toilet facility do members of your household usually use? Specify if other

1. Flush to septic tank
2. Pit latrine without slab/ Open pit
3. Other toilet type

What is the main material of the floor? Specify if other

1. Ceramic Tile
2. Other floor material

What is the main material of the exterior walls? Specify if other

1. Cement
2. Other

Livestock

How many of the following animals does this household own...

1. Cattle
2. Milk cows or bulls
3. Horses, donkeys or mules
4. Goats
5. Sheep

2. Tables of results used in Figures 1-3

Age-sex weighted prevalence of any vision impairment <6/12 and blindness, and cataract surgical coverage and effective cataract surgical coverage in the population 50 years and older disaggregated by four SEP measures.

Table S1. Weighted prevalence of any vision impairment and blindness and CSC/eCSC across the 2013 DHS EquityTool national scoring within the Gambia 2019 sample aged 50 years and older

	Quintile 1	Quintile 2	Quintile 3	Quintile 4	Quintile 5
**Population 50+**	N=814	N=794	N=804	N=804	N=804
**Any VI (%)**	28.9 (25.1-33.0)	24.3 (20.8-28.2)	27.1 (23.5-31.1)	27.7 (24.2-31.4)	27.7 (24.5-31.2)
**Blindness (%)**	2.7 (1.7-4.2)	2.7 (1.7-4.3)	1.4 (0.8-2.6)	2.2 (1.3-3.6)	3.0 (1.9-4.9)
**Cataract surgical threshold <6/60**					
**CSC (%)**	62.6 (51.5-72.4)	53.6 (41.0-65.7)	67.8 (57.4-76.7)	72.9 (61.2-82.1)	59.5 (44.3-73.0)
**eCSC (%)**	31.2 (22.3-41.6)	32.2 (22.2-44.2)	35.0 (23.6-48.3)	24.4 (14.1-38.7)	22.3 (13.7-34.1)
**Cataract surgical threshold <6/12**					
**CSC (%)**	32.4 (25.4-40.3)	30.5 (22.8-39.5)	38.6 (31.5-46.3)	33.6 (26.7-41.6)	33.8 (25.8-42.9)
**eCSC (%)**	15.2 (10.6-21.3)	17.0 (11.3-24.8)	19.2 (13.2-27.1)	11.7 (7.0-18.9)	13.3 (8.5-20.1)

Quintiles 1=Poorest, 5=richest

Table S2. Weighted prevalence of any vision impairment and blindness and CSC/eCSC in the bottom 40% and 20% of EquityTool national scoring within the Gambia 2019 sample aged 50 years and older

		Within-sample ET scoring			
	**All**	**Top 60%**	**Bottom 40%**	**Top 80%**	**Bottom 20%**
**Population 50+**	N=4020	N=2412	N=1608	N=3206	N=814
**Any VI (%)**	27.2 (25.4-29.0)	27.5 (25.4-29.7)	26.7 (23.8-29.7)	26.7 (24.8-28.7)	28.9 (25.1-33.0)
**Blind (%)**	2.4 (1.9-3.0)	2.2 (1.6-3.0)	2.7 (2.0-3.7)	2.3 (1.8-3.0)	2.7 (1.7-4.2)
**Cataract surgical threshold <6/60**					
**CSC (%)**	63.1 (57.6-68.3)	66.6 (59.2-73.2)	58.4 (50.2-66.2)	63.3 (56.9-69.2)	62.6 (51.5-72.4)
**eCSC (%)**	29.1 (24.2-34.6)	27.2 (20.9-34.6)	31.7 (24.8-39.5)	28.5 (23.0-34.8)	31.2 (22.3-41.6)
**Cataract surgical threshold <6/12**					
**CSC (%)**	33.9 (30.5-37.5)	35.5 (31.1-40.1)	31.6 (26.3-37.4)	34.3 (30.4-38.4)	32.4 (25.4-40.3)
**eCSC (%)**	15.3 (12.6-18.4)	14.8 (11.5-18.9)	16.0 (12.2-20.7)	15.3 (12.3-18.9)	15.2 (10.6-21.3)

Table S3. Weighted prevalence of any vision impairment and blindness and CSC/eCSC across five self-ranked positions on an economic ladder

	Step 1	Step 2	Step 3	Step 4	Step 5
**Population 50+**	N=227	N=1240	N=1919	N=489	N=112
**Any VI (%)**	16.5 (12.0-22.3)	20.6 (17.9-23.5)	32.6 (30.2-35.1)	27.9 (23.5-32.7)	21.3 (15.1-29.2)
**Blindness (%)**	3.1 (1.4-7.0)	2.0 (1.3-3.2)	2.9 (2.1-3.8)	1.2 (0.4-3.5)	2.4 (0.7-8.4)
**Cataract surgical threshold <6/60**					
**CSC (%)**	61.8 (35.8-82.4)	66.8 (56.9-75.4)	57.3 (50.0-64.3)	83.4 (65.5-93.0)	74.4 (25.4-96.1)
**eCSC (%)**	36.9 (19.2-59.0)	36.9 (27.6-47.4)	19.5 (14.2-26.2)	45.7 (27.3-65.4)	65.5 (23.9-92.0)

**Cataract surgical threshold <6/12**					
**CSC (%)**	32.2 (17.8-51.0)	37.2 (30.2-44.8)	32.2 (27.9-36.9)	33.4 (23.5-44.9)	36.0 (16.9-60.9)
**eCSC (%)**	19.2 (9.7-34.4)	20.2 (14.9-26.8)	11.0 (8.1-14.7)	18.1 (10.1-30.4)	26.1 (10.7-51.1)

Step 1=Poorest, 5=richest

Table S4. Weighted prevalence of any vision impairment and blindness and CSC/eCSC across three subjective food adequacy levels

	**All**		
	**Less than adequate**	**Just adequate**	**More than adequate**
**Population 50+**	N=976	N=3,006	N=37
**Any VI (%)**	37.4 (33.8-41.2)	24.2 (22.2-26.2)	2.2 (0.3-14.5)
**Blindness (%)**	3.7 (2.6-5.3)	2.0 (1.5-2.6)	2.2 (0.3-14.5)
**Cataract surgical threshold <6/60**			
**CSC (%)**	47.3 (38.6-56.1)	71.0 (64.6-76.7)	85.2 (36.0-98.3)
**eCSC (%)**	16.6 (10.3-25.7)	34.4 (28.1-41.4)	85.2 (36.0-98.3)
**Cataract surgical threshold <6/12**			
**CSC (%)**	28.4 (22.6-35.1)	35.6 (31.6-39.9)	87.3 (42.1-98.5)
**eCSC (%)**	9.9 (6.2-15.3)	16.8 (13.5-20.7)	87.3 (42.1-98.5)

Table S5. Weighted prevalence of any vision impairment and blindness and CSC/eCSC across three subjective income sufficiency levels

	**All**		
	**Less than adequate**	**Just adequate**	**More than adequate**
**Population 50+**	N=1,430	N=2,418	N=172
**Any VI (%)**	33.2 (30.3-36.2)	24.0 (21.9-26.3)	20.5 (14.3-28.4)
**Blindness (%)**	3.1 (2.3-4.3)	2.0 (1.5-2.7)	1.8 (0.7-4.8)
**Cataract surgical threshold <6/60**			
**CSC (%)**	53.1 (45.3-60.8)	71.2 (63.9-77.5)	78.7 (53.0-92.4)
**eCSC (%)**	20.2 (14.4-27.4)	34.0 (26.8-42.0)	72.5 (45.8-89.2)
**Cataract surgical threshold <6/12**			
**CSC (%)**	31.5 (26.2-37.2)	35.0 (30.4-39.8)	43.8 (24.9-64.7)
**eCSC (%)**	12.0 (8.6-16.4)	16.0 (12.4-20.3)	40.9 (22.5-62.2)
