# Supplementary material for: Socioeconomic position and eye health outcomes: identifying inequality in rapid population-based surveys
Source: BMJ Open. 2023 Mar 7;13(3):e069325. doi: 10.1136/bmjopen-2022-069325 (PMC10008479; doi:10.1136/bmjopen-2022-069325)
Supplement: Author's manuscript [file bmjopen-2022-069325.draft_revisions.pdf]

BMJ Open is committed to open peer review. As part of this commitment we make the peer review history of every article we publish publicly available.

When an article is published we post the peer reviewers' comments and the authors' responses online. We also post the versions of the paper that were used during peer review. These are the versions that the peer review comments apply to.

The versions of the paper that follow are the versions that were submitted during the peer review process. They are not the versions of record or the final published versions. They should not be cited or distributed as the published version of this manuscript.

BMJ Open is an open access journal and the full, final, typeset and author-corrected version of record of the manuscript is available on our site with no access controls, subscription charges or pay-per-view fees (http://bmjopen.bmj.com).

If you have any questions on BMJ Open's open peer review process please email info.bmjopen@bmj.com

BMJ Open

Socioeconomic position and eye health outcomes: identifying inequality in rapid population-based surveys

Journal:	*BMJ Open*
Manuscript ID	bmjopen-2022-069325
Article Type:	Original research
Date Submitted by the Author:	18-Oct-2022

Complete List of Authors:	McCormick, Ian; London School of Hygiene & Tropical Medicine, International Centre for Eye Health<br>Kim, Min; London School of Hygiene and Tropical Medicine Faculty of Infectious and Tropical Diseases<br>Hydara, Abba; Sheikh Zayed Regional Eye Care Centre<br>Olaniyan, Segun I; Sheikh Zayed Regional Eye Care Centre<br>Jobe, Modou; MRC Unit The Gambia at LSHTM<br>Badjie, Omar; Ministry of Health, Directorate of Health Promotion and Education<br>Sanyang, Nyakassi MB; Ministry of Finance, Gambia Bureau of Statistics<br>Jarju, Gibril; Ministry of Health, Directorate of Planning and Information<br>Njai, Modou; Ministry of Health, Directorate of Health Promotion and Education<br>Sankareh, Alhagie; Ministry of Health, Regional Directorate of Health Services, West Coast Health Region 1<br>Bastawrous, Andrew; London School of Hygiene & Tropical Medicine, International Centre for Eye Health<br>Allen, Luke; London School of Hygiene & Tropical Medicine, International Centre for Eye Health<br>Mactaggart, Islay; London School of Hygiene and Tropical Medicine, International Centre for Eye Health<br>Burton, Matthew J; London School of Hygiene and Tropical Medicine, International Centre for Eye Health; NIHR Moorfields Biomedical Research Centre<br>Ramke, Jacqueline; London School of Hygiene & Tropical Medicine, International Centre for Eye Health; The University of Auckland, School of Optometry and Vision Science

Keywords:	PUBLIC HEALTH, EPIDEMIOLOGY, OPHTHALMOLOGY

SCHOLARONE™
Manuscripts

I, the Submitting Author has the right to grant and does grant on behalf of all authors of the Work (as defined in the below author licence), an exclusive licence and/or a non-exclusive licence for contributions from authors who are: i) UK Crown employees; ii) where BMJ has agreed a CC-BY licence shall apply, and/or iii) in accordance with the terms applicable for US Federal Government officers or employees acting as part of their official duties; on a worldwide, perpetual, irrevocable, royalty-free basis to BMJ Publishing Group Ltd ("BMJ") its licensees and where the relevant Journal is co-owned by BMJ to the co-owners of the Journal, to publish the Work in this journal and any other BMJ products and to exploit all rights, as set out in our licence.

The Submitting Author accepts and understands that any supply made under these terms is made by BMJ to the Submitting Author unless you are acting as an employee on behalf of your employer or a postgraduate student of an affiliated institution which is paying any applicable article publishing charge ("APC") for Open Access articles. Where the Submitting Author wishes to make the Work available on an Open Access basis (and intends to pay the relevant APC), the terms of reuse of such Open Access shall be governed by a Creative Commons licence – details of these licences and which Creative Commons licence will apply to this Work are set out in our licence referred to above.

Other than as permitted in any relevant BMJ Author's Self Archiving Policies, I confirm this Work has not been accepted for publication elsewhere, is not being considered for publication elsewhere and does not duplicate material already published. I confirm all authors consent to publication of this Work and authorise the granting of this licence.

For peer review only

Title

Socioeconomic position and eye health outcomes: identifying inequality in rapid population-based surveys

Author list

Ian McCormick[1*]
Min J Kim[1]
Abba Hydara[2]
Segun I Olaniyan[2]
Modou Jobe[3]
Omar Badjie[4]
Nyakassi MB Sanyang[5]
Gibril Jarju[6]
Modou Njai[7]
Alhagie Sankareh[8]
Andrew Bastawrous[1]
Luke Allen[1]
Islay Z Mactaggart[1]
Matthew J Burton[1, 9]
Jacqueline Ramke[1, 10]

Affiliations

[1]International Centre for Eye Health, London School of Hygiene & Tropical Medicine, London, UK
[2]Sheikh Zayed Regional Eye Care Centre, Banjul, The Gambia
[3]Medical Research Council Unit The Gambia at London School of Hygiene & Tropical Medicine, Atlantic Boulevard, Fajara, The Gambia
[4]Non-Communicable Disease Programme, Directorate of Health Promotion and Education, Ministry of Health, Kotu, The Gambia
[5]Gambia Bureau of Statistics, Office of the Vice President, Kanifing, The Gambia
[6]Directorate of Planning and Information, Ministry of Health, Kotu, The Gambia
[7]Directorate of Health Promotion and Education, Ministry of Health, Kotu, The Gambia
[8]Regional Directorate of Health Services, Ministry of Health, West Coast Health Region 1, Kanifing, The Gambia
[9]National Institute for Health Research Biomedical Research Centre for Ophthalmology at Moorfields Eye Hospital NHS Foundation Trust and UCL Institute of Ophthalmology, London, UK
[10]School of Optometry and Vision Science, University of Auckland, Auckland, New Zealand

*Corresponding author

Ian McCormick
International Centre for Eye Health, London School of Hygiene & Tropical Medicine, Keppel Street, London, WC1E 7HT, UK
ian.mccormick@lshtm.ac.uk

Word count

4122

For peer review only - http://bmjopen.bmj.com/site/about/guidelines.xhtml
For peer review only

ABSTRACT

Objective: Monitoring health outcomes disaggregated by socioeconomic position (SEP) is crucial to ensure no one is left behind in efforts to achieve universal health coverage. In eye health planning, rapid population surveys are most commonly implemented; these need an SEP measure that is feasible to collect within the constraints of a streamlined examination protocol. We aimed to assess whether each of four SEP measures identified inequality – an underserved group or socioeconomic gradient – in key eye health outcomes.

Design: Population-based cross-sectional survey.

Participants: A subset of 4020 adults 50 years and older from a nationally representative sample of 9188 adults aged 35 years and older in The Gambia.

Outcome measures: Blindness (presenting visual acuity [PVA]<3/60), any vision impairment (VI) (PVA<6/12), cataract surgical coverage (CSC) and effective cataract surgical coverage (eCSC) at two operable cataract thresholds (<6/12 and <6/60) analysed by one objective asset-based measure (EquityTool) and three subjective measures of relative SEP (a self-reported economic ladder question and self-reported household food adequacy and income sufficiency).

Results: Subjective household food adequacy and income sufficiency demonstrated a socioeconomic gradient (queuing pattern) in point estimates of any VI and CSC and eCSC at both operable cataract thresholds. Any VI, CSC<6/60 and eCSC<6/60 were worse among people who reported inadequate household food compared to those with just adequate food. Any VI and CSC<6/60 were worse among people who reported not enough household income compared to those with just enough income. Neither the subjective economic ladder question nor the objective asset-wealth measure demonstrated any socioeconomic gradient or pattern of inequality in any of the eye health outcomes.

For peer review only

Conclusion: We recommend pilot-testing self-reported food adequacy and income sufficiency as SEP variables in vision and eye health surveys in other locations, including assessing the acceptability, reliability and repeatability of each question.

STRENGTHS AND LIMITATIONS

- Strengths included the use of a nationally representative sample of adults in a country typical of the low-and-middle income settings surveyed with rapid eye health survey methods
- Further, the outcomes we report are key eye health indicators included in WHO Eye Care Indicator Menu; effective cataract surgical coverage is one of two World Health Assembly endorsed indicators with a global target for 2030
- Also, we report both objective and subjective indicators of socioeconomic position to enable comparison
- Limitations include that the most recent objective asset wealth tool at the time of data collection was based on data collected six years prior (2013 DHS)
- In addition, our sample size was insufficient for analysis of intersectionality of socioeconomic position with other key equity dimensions

For peer review only

INTRODUCTION

The World Health Organization (WHO) and the *Lancet Global Health* Commission on Global Eye Health have called for vision and eye health data to be disaggregated by equity dimensions.[1, 2] This disaggregation is essential to understand eye health inequalities between population subgroups and to monitor progress towards eye health within Universal Health Coverage. Both WHO and the Commission identified socioeconomic position (SEP) as a key factor in eye health outcomes and the Commission recommended standard SEP indices be included in population-based vision impairment surveys.[1, 2]

Within countries, health inequalities exist along a socioeconomic gradient,[3, 4] and SEP indicators identify people experiencing poverty or deprivation via some means of social stratification.[5-7] SEP indicators can be used to measure the differential consequences of the social determinants of health and to monitor and evaluate policies and programmes designed to address inequity.[8-10] SEP can be described at an individual or group level (e.g., household or community) and can be measured in a variety of ways, such as using income, education, occupation, assets, nutrition, housing or a multidimensional index of such factors.[11] Measures can be objective or subjective, and absolute or relative.[11, 12] Poverty lines and asset wealth are objective measures, while self-reported, subjective measures of SEP assess individuals' perception of their own resources or standing relative to those living around them.

In comprehensive surveys of vision and eye health, lower SEP has been shown to be associated with higher prevalence of vision impairment and worse access to services,[13-16] though there is variation in SEP measures used. The Rapid Assessment of Avoidable Blindness (RAAB) is the most commonly conducted rapid vision survey worldwide.[17, 18] RAAB has streamlined examination protocols compared to comprehensive surveys, but generates key outputs that include the prevalence of vision impairment and effective cataract surgical coverage (eCSC) – both important indicators for monitoring global eye health.[19, 20] Currently, age and gender are the only equity dimensions routinely recorded in

For peer review only

RAAB while a disability module is optional. To expand data collection to include a measure of SEP within RAAB it is crucial that steps are taken to identify which measures are feasible to collect regularly and might identify those most at risk of being left behind. The anticipated 'time-cost' of incorporating SEP indicators into a rapid survey means more concise questions need to be considered.

In this paper, we aimed to assess whether one objective asset-based measure and three subjective measures of relative SEP demonstrated a socioeconomic gradient or inequality in key eye health outcomes in the population 50 years and older using data from the 2019 Gambia National Eye Health Survey, a comprehensive cross-sectional survey with an embedded RAAB.[21] Further, we aimed to assess the level of association between the objective asset based measure and three subjective measures of relative SEP and recommend one or more indicators to pilot test in other locations.

METHODS

Data collection and categorisation

A detailed summary of the 2019 Gambia National Eye Health Survey sampling and examination protocol is provided elsewhere.[22] Briefly, the 2013 National Census was used as a sampling frame to select a nationally representative sample of adults aged 35 years and older in clusters of 30 people. A multi-stage, stratified, cluster random sampling with probability proportionate to size strategy was used. The sample was stratified by historic geographic regions of the country and the urban and rural population proportions within them, using The Gambia Bureau of Statistics' definitions of urban and rural.

At the individual level, sociodemographic data collected included age, sex, marital status, religion, ethnicity, education, occupation and subjective socioeconomic position (details below). A questionnaire on asset ownership was completed at the household level via the household head or an adult household key informant (details below). No absolute measure of income or expenditure was collected. Distance visual acuity (VA) was measured indoors at 3 metres using the Peek Acuity

For peer review only - http://bmjopen.bmj.com/site/about/guidelines.xhtml

application on Huawei MediaPad M3 tablet devices.[23] Lens grading was done on slit-lamp examination following dilation with tropicamide 1% eye drops and according to the WHO Cataract Grading Tool.[24] The survey was completed by four data collection teams between February and July 2019.

Eye health outcomes

We report blindness and vision impairment according to existing WHO definitions. Cataract surgical coverage (CSC) and effective cataract surgical coverage (eCSC) were calculated using the most recent definition.[25] For the purposes of determining service coverage, cataract classified as gradable, mature or hypermature met the definition of *operable*. *Operated* cataract was defined where aphakia (excluding cases of couching) or an intraocular lens was recorded. CSC and eCSC were reported at two best-corrected VA surgical thresholds: <6/12 and <6/60, and the threshold for effective coverage (post-operative presenting VA) was ≥6/12.[19]

Box 1. Four key eye health outcomes and their descriptions.

Eye health outcome	Description
Blindness	Presenting VA (i.e., with available correction, if worn) in the better eye worse than 3/60
Any vision impairment (VI)	Presenting VA (i.e., with available correction, if worn) in the better eye worse than 6/12
Cataract surgical coverage (CSC)	The number of people with operated cataract as a proportion of those having operable plus operated cataract
Effective cataract surgical coverage (eCSC)	The number of people with operated cataract and a good visual outcome (presenting VA 6/12 or better) as a proportion of those having operable plus operated cataract

SEP indicators

For peer review only - http://bmjopen.bmj.com/site/about/guidelines.xhtml

SEP was assessed in four separate ways. The first was to divide the study population into SEP quintiles using the 'EquityTool' analysis provided by Metrics for Management (www.m4mgmt.org). EquityTool is an *objective relative SEP measure* that uses a short asset ownership and household characteristics questionnaire to generate wealth quintiles with a minimum level of agreement (Kappa ≥0.75) with quintiles derived from underlying (more comprehensive) Demographic and Health Survey (DHS) data. The EquityTool for The Gambia used the 2013 DHS and consisted of 16 questions (appendix p 1).[26] Participants were assigned to a national wealth quintile benchmarked to the 2013 DHS according to their household questionnaire score. In addition to quintile assignment, the EquityTool output includes three continuous variables for national, urban and rural scoring on the questionnaire. We created evenly distributed within-sample quintiles based on the national score for all participants. These were not benchmarked to national thresholds and represented the distribution of asset wealth within the sample only.

For the remaining three questions we used *subjective relative SEP measures* relating to food adequacy, income sufficiency and self-perceived position on an economic ladder (Box 2). These questions come from Howe and colleagues' analysis of 11,280 Malawian households that found them to be better at identifying low-income households (those living on less than US$1/ day) than a composite wealth index.[8]

Box 2. Three subjective socioeconomic position variables including the question and possible responses.

Food adequacy	"When you think about the food in your household would you say you have 1) Less than adequate food for the needs of your household, 2) Just adequate food for the needs of your household or 3) More than adequate food for the needs of your household."
Income sufficiency	"When you think about the income in your household would you say it is 1) Not enough to cover our needs, we must borrow, 2) Not enough to cover our

For peer review only

	needs, we use savings, 3) Just enough to cover our needs, 4) Enough to cover our needs, we are able to save a little or 5) Enough to cover our needs, we are building savings."
Economic ladder question	"Please look at this ladder [5 step ladder pictured]. If the bottom step (1) of the ladder represents the people who are poorest in your community, and the top step (5) represents the people who are richest, on which step of the ladder do you feel your household stands on?"

Patterns of inequality

We sought to identify which of the patterns of inequality described in the WHO *Handbook on Health Inequality Monitoring*, were present for our outcomes, namely, mass deprivation, marginal exclusion, queuing or complete coverage.[9] Mass deprivation describes poor health outcomes in all SEP groups except the richest. Marginal exclusion describes worse outcomes in the poorest group compared to all other. Queuing describes an approximately linear decrease in outcomes down SEP groups. Complete coverage describes universally good outcomes across SEP groups.

Data analysis

We assessed the association between the ordinal categories of objective relative asset wealth and each subjective SEP measure by estimating Kendall's tau-b correlation coefficient (assuming a monotonic relationship for all three comparisons). Kendall's tau-b coefficient values range from -1 to 1, with values closer to 1 indicating a stronger relationship and a negative value indicating an inverse relationship.

All analyses were done in Stata 16. The prevalence of any VI, blindness, CSC and eCSC and their 95% confidence intervals were calculated adjusted for clustering and post-stratified (weighted) according to the age-sex distribution of The Gambia 2013 Census using the svy command.

For peer review only

In reporting selected eye health outcomes, we restricted our population of interest to people 50 years and older as this age group is directly relevant to the RAAB survey methodology.

Missing data

To prevent listwise deletion during analyses, raw data were checked for completeness. In August 2020, follow-up data collection was completed by telephone to address some missing data and following this, forms with persisting missing critical data were deleted from the final sample.[22] EquityTool questions were imputed for 398 participants (4.3%) in the final dataset, whereby missing values in a cluster were replaced with the most frequent, non-missing value from within the cluster. Missing values were not substituted for questions that had more than one “most frequent” response in the cluster (e.g., bimodal with equal numbers of observed values for “yes” and “no”).[21]

Patient and public involvement

Survey participants were not involved in the design of the survey.

RESULTS

There were 9,188 participants examined out of 11,027 enumerated (response rate 83%). The mean age of the sample was 49.6 years (standard deviation 13.4), 71% were female and 55% were living in an urban location. There were 4020 participants aged 50 years or older included in the sample. The sociodemographic and socioeconomic characteristics of the subgroup aged 50 years and older are presented in Table 1.

Table 1. Sociodemographic and socioeconomic position characteristics in the population 50 years and older, The Gambia National Eye Health Survey, 2019.

	Population 50 years and older (N=4020)		
	**N**	**Crude %**	**Weighted %**

For peer review only

**Sex**			
Male	1,576	39.2	50.9
Female	2,444	60.8	49.1
**Marital status**			
Married/ cohabiting	2,912	72.4	76.3
Widowed	1,035	25.8	21.8
Divorced/ separated	57	1.4	1.4
Never married	16	0.4	0.5
**Location**			
Urban	2,180	54.2	54.8
Rural	1,840	45.8	45.2
**Education level**			
Non-formal (Quranic)[a]	2,455	61.1	58.9
Pre-school[b]/ no school	823	20.5	20.4
Primary[b]	229	5.7	6.1
Secondary[b]/ vocational	318	7.9	9.5
Higher	86	2.1	2.7
Don't know	109	2.7	2.4
**Occupation category**			
Unemployed	910	22.6	21.4
Manual/ unskilled	1,987	49.4	47.8
Trade/ skilled manual	714	17.8	19.4
Professional	120	3.0	3.9
Retired	238	5.9	5.9
Other	51	1.3	1.6
**National asset wealth quintiles (2013 DHS EquityTool)**			
Q1 - poorest	366	9.1	9.4
Q2	547	13.6	14.1
Q3	1,022	25.4	25.3
Q4	936	23.3	24.0
Q5 - richest	1,149	28.6	27.3

For peer review only - http://bmjopen.bmj.com/site/about/guidelines.xhtml

**Within-sample asset wealth quintiles**			
Q1 - poorest	814	20.3	-
Q2	794	19.8	-
Q3	804	20.0	-
Q4	804	20.0	-
Q5 - richest	804	20.0	-
**Food adequacy**			
Less than adequate	976	24.3	24.3
Just adequate	3,006	74.8	74.7
More than adequate	37	0.9	1.0
Missing	1	0.0	-
**Income sufficiency**			
Not enough, must borrow	922	22.9	22.6
Not enough, use savings	508	12.6	13.2
Just enough	2,418	60.2	59.9
Enough, save a little	167	4.2	4.1
Enough, build savings	5	0.1	0.1
Missing	0	0.0	-
**Economic ladder question (ELQ)**			
Step 1 - poorest	227	5.7	5.5
Step 2	1,240	30.9	29.5
Step 3	1,919	47.7	49.3
Step 4	489	12.2	12.8
Step 5 - richest	112	2.8	3.0
Missing	33	0.8	-

[a]Non-formal (Quranic) describes a system where students study mostly religious education at home for a non-specific number of years without any formal curriculum; [b]Includes Madrassa and non-Madrassa systems (Madrassa describes schools where Arabic is the medium of instruction and with emphasis on Islamic education)

There was an uneven distribution of survey participants across the national EquityTool quintiles generated from the 2013 DHS. The poorest two quintiles were under-represented (only 9% of the

sample were in the poorest quintile [Q1] and 14% were in Q2) while the wealthiest three quintiles were over-represented (Table 1). For all subsequent analyses, in place of these groups, we used the evenly distributed within-sample asset wealth quintiles that we generated. Very few participants placed themselves in the wealthiest category of any of the three subjective measures. For subsequent analyses we combined the two highest categories of income sufficiency because of the very small number of participants in the wealthiest group and combined the poorest two groups to leave three levels, consistent with the food adequacy measure. Over half the participants had received a non-formal (Quranic) education; this did not allow for an ordinal scale of education levels to be compared with other SEP measures and education was not included in the analyses.

Relationship between objective and subjective SEP measures in the sample 50 years and older

For the population 50 years and older, the within-sample asset wealth quintiles showed weak positive correlation with each of the subjective food adequacy, income sufficiency and economic ladder question responses (Kendall's tau-b statistic (τ_b) = 0.09, 0.15 and 0.18 respectively; N=4019, 4020 and 3987 respectively; p<0.001 for all three estimates).

Key eye health outcomes among the population 50 years and older

Among the total population 50 years and older, the age-sex weighted prevalence of blindness was 2.4% (95% confidence interval [CI] 1.9-3.0%) and of any VI was 27.2% (95% CI 25.4-29.0%). In the same population, the age-sex weighted CSC and eCSC were 63.1% (95% CI 57.6-68.3%) and 29.1% (24.2-34.6%) respectively at the <6/60 threshold for cataract surgery. At the lower threshold for cataract surgery of <6/12, the CSC and eCSC estimates were 33.9% (30.5-37.5%) and 15.3% (12.6-18.4%) respectively.

Weighted prevalence of vision impairment and cataract surgical coverage in the population 50 years and older disaggregated by SEP measures

Objective relative asset wealth (EquityTool)

For peer review only

No pattern of inequality was identified across within-sample wealth quintiles for any VI, blindness, CSC or eCSC at either threshold and there were no statistically significant differences between any of the quintiles (Figures 1-3 and appendix Table S1). Consolidating within-sample quintiles to a binary comparison of asset wealth groups showed the weighted prevalence of any VI and blindness and weighted coverage (by any measure) in the poorest one or two quintiles was not significantly different to the top four or three respectively, or to the overall weighted prevalence or coverage estimates (appendix Table S2).

Fig 1. Age-sex weighted prevalence of blindness and any vision impairment in the population 50 years and older by four socioeconomic position variables, The Gambia 2019

Fig 2. Age-sex weighted cataract surgical coverage and effective cataract surgical coverage (cataract surgical threshold <6/60) in the population 50 years and older by four socioeconomic position variables, The Gambia 2019

Fig 3. Age-sex weighted cataract surgical coverage and effective cataract surgical coverage (cataract surgical threshold <6/12) in the population 50 years and older by four socioeconomic position variables, The Gambia 2019

Subjective measure: Economic Ladder Question

Almost half of participants placed themselves on the middle position of the ladder (step 3); three quarters of responses were 'clumped' together across steps 2 and 3 (Table 1). There was no pattern of inequality in any VI, blindness, CSC or eCSC across the five economic ladder steps. Step 3 (the largest group) had a significantly higher prevalence of any VI compared to steps 1, 2 and 5 while there were no significant differences in blindness prevalence across the ladder (Figure 1). There were few participants or cataract cases placed at either end of the scale and confidence intervals for the estimates in these positions were wide, however, $eCSC_{<6/60}$ was significantly higher for people who placed themselves on step 2 compared to step 3 (Figure 2). See appendix Table S3.

For peer review only - http://bmjopen.bmj.com/site/about/guidelines.xhtml

Subjective measure: Household food adequacy

Only 1% of people reported having more than adequate household food, while 24% reported less than adequate household food (Table 1). Considering the point estimates, a queuing pattern of inequality was apparent across food adequacy levels for almost all eye health outcomes, however, confidence intervals for the estimates in the top group were very wide. Any VI, blindness, $CSC_{<6/60}$ and $eCSC_{<6/60}$ were all significantly worse among those with less than adequate food compared to those with just adequate food (Figures 1 & 2) while the difference between the two was not significant for $CSC_{<6/12}$ and $eCSC_{<6/12}$ (Figure 3). See appendix Table S4.

Subjective measure: Household income sufficiency

After consolidating the original five levels of income sufficiency to three, still only 4% of people reported ‘more than enough’ household income to be able to ‘save a little’ (level 4) or ‘build savings’ (level 5). Combined, 36% of participants occupied the two original ‘not enough’ levels of household income (‘must borrow’ and ‘use savings’). See appendix Table S5. Considering the point estimates, a queuing pattern of inequality was seen across three levels of income sufficiency for all eye health outcomes. As for food adequacy, the low numbers of participants and cataract cases in the top group meant confidence intervals were very wide, but there were significant differences in any VI and $CSC_{<6/60}$ between the middle and bottom groups (Figures 1 & 2).

For peer review only

DISCUSSION

The 2019 Gambia National Eye Health Survey estimated key eye health outcomes in a nationally representative sample of adults aged 35 years and older and included one objective measure of asset-based relative wealth (EquityTool) and three subjective measures of socioeconomic position. We analysed data on a sub-group of 4020 adults aged 50 years and older.

The Gambia's 2015-16 Integrated Household Survey estimated an absolute measure of poverty (poverty line) based on per capita consumption. The proportion of the population living in poverty was 48.6%, an increase of 0.5% since 2010.[27] While an absolute measure like this may be a more intuitive concept of poverty, we focussed on relative SEP measures as they identify inequality in all settings (including where absolute poverty decreases with development) and are well aligned with the aim of monitoring eye health within UHC.

We found no patterns of inequality or significant differences between quintiles for any of our selected eye health outcomes by relative objective asset wealth. Similarly, a 2020 RAAB survey in rural Nigeria included an EquityTool questionnaire benchmarked to 2013 national DHS data.[28] The proportion of participants in each quintile was not reported, but there was no pattern of inequality or significant differences in blindness, severe VI or moderate VI across wealth quintiles. Asset-based measures such as EquityTool are easier to capture than income or expenditure, particularly in LMIC, and participant responses can often be verified at interview.[8] However, certain assets may have a different relationship with SEP in different subgroups, which must be accounted for in analyses and interpretation. Even then, asset ownership does not distinguish between asset quality and assets may not reflect the short-term resource availability often required to make out-of-pocket payments for health care.[29] In addition, wealth indices may conflate community-level factors with household-level factors,[8] and where national thresholds are used in a relatively homogenous sub-national region, as can be surveyed with RAAB, respondents may be 'clumped' in one or two quintiles.[30] From the perspective of a rapid survey methodology, multiple additional questions per individual or household

For peer review only - http://bmjopen.bmj.com/site/about/guidelines.xhtml

For peer review only

may increase the time and cost of data collection to the point where it becomes a barrier to eye health planners embarking on a survey.

Howe et al. proposed three desirable features of an SEP indicator for use in health research or evaluation in LMIC: that it is feasible in terms of cost and complexity, reliable and reproducible and that the underlying social stratification process be understood.[8] In terms of their simplicity, any of the three subjective SEP measures reviewed here would be feasible to incorporate into a rapid survey protocol and the stratification generated by each is straightforward.

The subjective economic ladder question did not identify any pattern of inequality in outcomes across the five steps. In the two most frequently selected positions (steps 2 and 3), any VI and $eCSC_{<6/60}$ were significantly better in the poorer of the two SEP positions. However, subjective socioeconomic position has been shown to be a useful predictor of health in older people elsewhere,[31] and the economic ladder question may be worth reassessing in vision and eye health surveys in other settings. We presented an image of a ladder with five steps. Participants may have been distributed differently across a nine- or ten-step ladder (as has been used in other studies[32, 33]) which may have shown a pattern of inequality not identified here.

The three-tiered subjective measures of household food adequacy and income sufficiency demonstrated that very few people in The Gambia self-identify as having more than enough of either resource. The concept of food security encompasses the need to have the physical or financial means to obtain quality food on a regular and sustained basis.[34] In LMIC, food has been shown to be households' single biggest expense,[35] and food inadequacy may be a useful tool to identify the people with fewest resources in such settings. In 2015-16, food accounted for almost 60% of total household consumption expenditure in The Gambia.[27] A direct, subjective measure of food adequacy may be better than objective measures of adequacy, such as consumption or body mass index, given natural variations in physiology and the absence of agreed norms.[36] Historically, The Gambia has suffered from food insecurity, with levels increasing in recent years on account of climate-related flood and

For peer review only - http://bmjopen.bmj.com/site/about/guidelines.xhtml

For peer review only

drought.[37] Almost a quarter of participants aged 50 years and older reported less than adequate household food and people who self-identified in this bottom group experienced worse eye health outcomes than those in the middle group. Outcomes were best in the top group but confidence intervals for the estimates were very wide due to the very small group size. Food poverty is also prevalent in high-income countries and also associated with households' financial resources.[34] In high and some middle income settings, the smallest group may commonly be the 'less than adequate' group rather than the 'more than adequate' seen here. In these settings, a binary comparison can be made between 'more than adequate' and 'just adequate' in place of 'just adequate' and 'less than adequate'.

Subjective income sufficiency has a complex relationship with actual income and subjective well-being and may vary over the life course.[38] In a study of people 50 years and older across twelve high-income countries it was most strongly associated with individuals' net worth and employment status, but may be over-estimated in the oldest age groups.[38] In The Gambia, just over a third of participants aged 50 years and older reported insufficient household income such that the household had to borrow money or spend savings to meet their needs. Eye health outcomes stratified by self-reported household income sufficiency followed a similar trend to food adequacy. Point estimates were all lowest in the bottom group, with any VI and $CSC_{<6/60}$ outcomes significantly worse than the middle group.

The distribution of responses to food adequacy and income sufficiency limited the extent to which any pattern of inequality could be reliably interpreted as the measures were effectively reduced to binary "not enough" vs "just enough" comparisons. However, as potential eye health equity indicators, they were able to demonstrate that people who perceived themselves to have the least resources were significantly worse-off for any VI prevalence, and CSC and eCSC at the <6/60 threshold. In The Gambia, eCSC was low in the overall population 50 years and older at both thresholds ($eCSC_{<6/60}$ 30%; $eCSC_{<6/12}$ 15%). Point estimates for those with insufficient income were lower (21% and 12% respectively) and lower again for inadequate food (16% and 10% respectively). These subjective, experience-based SEP

For peer review only - http://bmjopen.bmj.com/site/about/guidelines.xhtml

measures may capture less obvious aspects of social status and allow research participants to be centred in the process of identifying socioeconomic inequality in each survey setting.[8] Tracking eCSC in socioeconomic groups stratified by food adequacy and/ or income sufficiency may be a useful way to monitor eye health equity within countries over time and is aligned with WHO's emphasis on countries achieving a 30 percentage points increase in all population sub-groups. Unlike SEP quintiles, the relative size of the self-reported groups may increase or decrease over time and, alongside changing effective coverage, this information may also be useful for national and sub-national service planning.

The objective relative asset wealth metric showed weak positive association with each of the three subjective SEP measures used. Varying levels of agreement between objective and subjective SEP metrics have been reported in the literature. In eye health, a cataract vision impairment and poverty case-control study across three low- and middle-income countries found that measures of per capita expenditure, asset ownership and self-rated wealth were highly correlated.[35] A trachoma and poverty case-control study in Ethiopia found moderate agreement between a within-study asset-wealth index and self-rated wealth.[39] A 2004-2005 national household survey in Malawi found poor agreement between an objective asset-wealth index and the same subjective SEP measures we used here and the authors noted that the two approaches appeared to be measuring different concepts.[8] Individual indicators of SEP may be correlated but are rarely proxies for each other as they variously represent unique and shared aspects of the concept.[40] However, to identify a socioeconomic group at risk of – or demonstrate a socioeconomic gradient in – a poor health outcome such as vision impairment, the choice of relative SEP indicator may not be crucial.[8]

RAAB surveys can be nationally representative but have more often been carried out at the subnational level, where participants' place of residence is often either predominantly rural or urban. We considered our sample size of around 4000 people 50 years and older too small to examine the effect of intersectionality on eye health outcomes and did not report outcomes by SEP in urban and

For peer review only - http://bmjopen.bmj.com/site/about/guidelines.xhtml

rural populations separately. This is of relevance for rare outcomes such as blindness and cataract service coverage indicators where the number of observations in subgroups may be small. However, intersectionality has been shown to be an important consideration in eye health,[41] and disaggregation by two equity dimensions may be feasible for some outcomes in larger RAAB surveys. Further research is required to address this question in future surveys. Another consideration for future research is the use of small-area deprivation indices such as those already employed in some high-income settings.[42] Pre-existing metrics could be applied at the cluster level and be used alongside or instead of individual level SEP data.

Our study had several limitations. Our sample was not evenly distributed across the quintiles of asset wealth generated by the EquityTool based on the 2013 DHS. Another DHS was completed in The Gambia in 2019-2020 but the subsequent EquityTool was unavailable at the time of our survey. The questions in the EquityTool elicit durable asset ownership and household characteristics. Given six years had passed between the 2013 DHS on which the Equity Tool was based and our survey, we assume ongoing urbanisation and improved water and sanitation likely contributed to the under-representation of lower SEP quintiles and over-representation of higher SEP quintiles in our sample.[43] There may have been stronger associations between participant responses to the subjective SEP questions and a more contemporary or comprehensive asset wealth index. We did not assess the reliability or repeatability of the subjective SEP measures in this study, nor did we assess their acceptability in the population; however, previous research in a low-income population in the USA indicates self-reporting subjective income sufficiency may be less contentious than reporting a specific income level.[44] The wording of the questions may need to be refined in different settings to ensure they make sense to the population. These issues should all be addressed in future research. A comparison of the effects of different SEP dimensions (including those not described here) on VI status may help identify which specific socioeconomic characteristics or resources are most important for good eye health outcomes. We did not try to estimate the effects of various SEP measures on e.g.,

For peer review only - http://bmjopen.bmj.com/site/about/guidelines.xhtml

vision impairment prevalence. Any such analysis of multiple SEP indicators requires careful causal interpretation to avoid mutual adjustment fallacies, and was beyond the aims of this study.[40]

We propose pilot testing the inclusion of two subjective SEP indicators – food adequacy and income sufficiency – in future RAAB surveys. Both SEP indicators demonstrated an inequality gradient in eye health outcomes in the population 50 years and older and may be useful for tracking progress in eCSC among population groups most in need of services. These indicators should be tested in a variety of countries with different income levels and the acceptability of each question should be reviewed in different cultural contexts.

COMPETING INTERESTS

None declared.

FUNDING

This research was funded by The Queen Elizabeth Diamond Jubilee Trust [Grant number TG002]. MJB is supported by the Wellcome Trust [207472/Z/17/Z].

AUTHOR CONTRIBUTIONS

JR conceptualised the research question. IM, MJK, AH, SIO, MJ, AB, IZM, MJB and JR contributed to the design and conduct of the survey. IM, MJK and IZM analysed the data. OB, NMBS, GJ, MN, AS and LA contributed to interpreting the results. IM drafted the original manuscript. All co-authors reviewed and edited the manuscript.

ACKNOWLEDGEMENT

JR's position at the University of Auckland is funded by the Buchanan Charitable Foundation, New Zealand.

For peer review only

ETHICS APPROVAL

Ethical approval for the study was granted by The Gambia Government/Medical Research Council Joint Ethics and Scientific Coordinating Committee (Ref SCC 1635) and the London School of Hygiene & Tropical Medicine Research Ethics Committee (Ref 16172).

DATA SHARING STATEMENT

The anonymised dataset will be made available on reasonable request from the study team.

For peer review only

References

1. World Health Organization, *World report on vision*. 2019: Geneva.
2. Burton, M.J., Ramke, J., Marques, A.P., et al., *The Lancet Global Health Commission on Global Eye Health: vision beyond 2020.* The Lancet Global Health, 2021. **9**(4): p. e489-e551.
3. Marmot, M., *Social determinants of health inequalities.* The Lancet, 2005. **365**(9464): p. 1099-1104.
4. Marmot, M., Friel, S., Bell, R., Houweling, T.A.J., and Taylor, S., *Closing the gap in a generation: health equity through action on the social determinants of health.* The Lancet, 2008. **372**(9650): p. 1661-1669.
5. Galobardes, B., Shaw, M., Lawlor, D.A., Lynch, J.W., and Davey Smith, G., *Indicators of socioeconomic position (part 1).* Journal of Epidemiology and Community Health, 2006. **60**(1): p. 7.
6. Galobardes, B., Shaw, M., Lawlor, D.A., Lynch, J.W., and Davey Smith, G., *Indicators of socioeconomic position (part 2).* Journal of epidemiology and community health, 2006. **60**(2): p. 95-101.
7. Galobardes, B., Lynch, J., and Smith, G.D., *Measuring socioeconomic position in health research.* British Medical Bulletin, 2007. **81-82**(1): p. 21-37.
8. Howe, L.D., Hargreaves, J.R., Ploubidis, G.B., De Stavola, B.L., and Huttly, S.R., *Subjective measures of socio-economic position and the wealth index: a comparative analysis.* Health Policy Plan, 2011. **26**(3): p. 223-32.
9. World Health Organization, *Handbook on health inequality monitoring: with a special focus on low-and middle-income countries*. 2013: World Health Organization.
10. World Health Organization, *A conceptual framework for action on the social determinants of health.* 2010.
11. Krieger, N., Williams, D.R., and Moss, N.E., *Measuring social class in US public health research: concepts, methodologies, and guidelines.* Annu Rev Public Health, 1997. **18**: p. 341-78.
12. Conway, D.I., McMahon, A.D., Brown, D., and Leyland, A.H., *Measuring socioeconomic status and inequalities*, in *Reducing social inequalities in cancer: evidence and priorities for research*. 2019: Lyon, France.
13. Gilbert, C.E., Shah, S.P., Jadoon, M.Z., et al., *Poverty and blindness in Pakistan: results from the Pakistan national blindness and visual impairment survey.* BMJ (Clinical research ed.), 2008. **336**(7634): p. 29-32.
14. Tafida, A., Kyari, F., Abdull, M.M., et al., *Poverty and Blindness in Nigeria: Results from the National Survey of Blindness and Visual Impairment.* Ophthalmic Epidemiology, 2015. **22**(5): p. 333-341.
15. Bourne, R.R., Dineen, B.P., Huq, D.M., Ali, S.M., and Johnson, G.J., *Correction of refractive error in the adult population of Bangladesh: meeting the unmet need.* Invest Ophthalmol Vis Sci, 2004. **45**(2): p. 410-7.
16. Braithwaite, T., Verlander, N.Q., Peto, T., et al., *National Eye Survey of Trinidad and Tobago (NESTT): prevalence, causes and risk factors for presenting vision impairment in adults over 40 years.* British Journal of Ophthalmology, 2020. **104**(1): p. 74-80.
17. Mactaggart, I., Limburg, H., Bastawrous, A., Burton, M.J., and Kuper, H., *Rapid Assessment of Avoidable Blindness: looking back, looking forward.* The British journal of ophthalmology, 2019. **103**(11): p. 1549-1552.
18. Mactaggart, I., Wallace, S., Ramke, J., et al., *Rapid assessment of avoidable blindness for health service planning.* Bulletin of the World Health Organization, 2018. **96**(10): p. 726.
19. Keel, S., Müller, A., Block, S., et al., *Keeping an eye on eye care: monitoring progress towards effective coverage.* The Lancet Global Health, 2021.

For peer review only

20. McCormick, I., Mactaggart, I., Resnikoff, S., et al., *Eye health indicators for universal health coverage: results of a global expert prioritisation process.* British Journal of Ophthalmology, 2021: p. bjophthalmol-2020-318481.
21. Hydara, A., Bastawrous, A., Bell, S., et al., *The Gambia National Eye Health Survey 2019: survey protocol [version 2; peer review: 2 approved].* Wellcome Open Research, 2021. **6**(10).
22. Hydara, A., Bastawrous, A., Bell, S., et al., *The Gambia National Eye Health Survey 2019: survey protocol [version 1; peer review: 1 approved with reservations].* Wellcome Open Research, 2021. **6**(10).
23. Bastawrous, A., Rono, H.K., Livingstone, I.A., et al., *Development and Validation of a Smartphone-Based Visual Acuity Test (Peek Acuity) for Clinical Practice and Community-Based Fieldwork.* JAMA Ophthalmol, 2015. **133**(8): p. 930-7.
24. Thylefors, B., Chylack, L.T., Jr., Konyama, K., et al., *A simplified cataract grading system.* Ophthalmic Epidemiol, 2002. **9**(2): p. 83-95.
25. McCormick, I., Butcher, R., Evans, J.R., et al., *Effective cataract surgical coverage in adults aged 50 years and older: estimates from population-based surveys in 55 countries.* The Lancet Global Health, 2022.
26. Metrics for Management. *Gambia EquityTool*. 2018; Available from: equitytool.org.
27. Gambia Bureau of Statistics. *Integrated Household Survey 2015/16: Vol III Prevalence and depth of poverty*. 2017 [cited 2021 12.10]; Available from: https://www.gbosdata.org/downloads/integrated-household-survey-ihs-24.
28. Gascoyne, B., Jolley, E., Ogundimu, K., Chailloux, A., and Owoeye, J. *Rapid Assessment of Avoidable Blindness (RAAB) report, Kogi State, Nigeria*. 2020 [cited 2021 19.10]; Available from: https://research.sightsavers.org/wp-content/uploads/sites/8/2021/03/Rapid-Assessment-of-Avoidable-Blindness-RAAB-report-Kogi-State-Nigeria-IF-October-2020.pdf.
29. Vyas, S. and Kumaranayake, L., *Constructing socio-economic status indices: how to use principal components analysis.* Health Policy Plan, 2006. **21**(6): p. 459-68.
30. Jolley, E., Buttan, S., Engels, T., et al., *Prevalence of Visual Impairment and Coverage of Cataract Surgical Services: Associations with Sex, Disability, and Economic Status in Five Diverse Sites.* Ophthalmic Epidemiology, 2020. **27**(6): p. 429-437.
31. Demakakos, P., Nazroo, J., Breeze, E., and Marmot, M., *Socioeconomic status and health: The role of subjective social status.* Social Science & Medicine, 2008. **67**(2): p. 330-340.
32. Adler, N.E., Epel, E.S., Castellazzo, G., and Ickovics, J.R., *Relationship of subjective and objective social status with psychological and physiological functioning: Preliminary data in healthy, White women.* Health psychology, 2000. **19**(6): p. 586.
33. Ravallion, M. and Lokshin, M., *Subjective economic welfare*. Vol. 2106. 1999: World Bank Publications.
34. Gorton, D., Bullen, C.R., and Mhurchu, C.N., *Environmental influences on food security in high-income countries.* Nutrition Reviews, 2010. **68**(1): p. 1-29.
35. Kuper, H., Polack, S., Eusebio, C., et al., *A case-control study to assess the relationship between poverty and visual impairment from cataract in Kenya, the Philippines, and Bangladesh.* PLoS Med, 2008. **5**(12): p. e244.
36. Mahadevan, R. and Hoang, V., *Is There a Link Between Poverty and Food Security?* Social Indicators Research, 2016. **128**(1): p. 179-199.
37. World Food Programme. *Gambia Country Profile*. 2021 [cited 2021 23.06]; Available from: https://www.wfp.org/countries/gambia.
38. Litwin, H. and Sapir, E.V., *Perceived income adequacy among older adults in 12 countries: findings from the survey of health, ageing, and retirement in Europe.* Gerontologist, 2009. **49**(3): p. 397-406.
39. Habtamu, E., Wondie, T., Aweke, S., et al., *Trachoma and Relative Poverty: A Case-Control Study.* PLoS neglected tropical diseases, 2015. **9**(11): p. e0004228-e0004228.

For peer review only

40. Green, M.J. and Popham, F., *Interpreting mutual adjustment for multiple indicators of socioeconomic position without committing mutual adjustment fallacies.* BMC Public Health, 2019. **19**(1): p. 10.
41. Ramke, J., Kyari, F., Mwangi, N., et al., *Cataract Services are Leaving Widows Behind: Examples from National Cross-Sectional Surveys in Nigeria and Sri Lanka.* International Journal of Environmental Research and Public Health, 2019. **16**(20): p. 3854.
42. Exeter, D.J., Zhao, J., Crengle, S., Lee, A., and Browne, M., *The New Zealand Indices of Multiple Deprivation (IMD): A new suite of indicators for social and health research in Aotearoa, New Zealand.* PLOS ONE, 2017. **12**(8): p. e0181260.
43. Gambia Bureau of Statistics (GBoS) and ICF. *The Gambia Demographic and Health Survey 2019-20: Chapter 2 Housing characteristics and household population*. 2021 [cited 2021 11.10]; Available from: https://dhsprogram.com/pubs/pdf/FR369/FR369.pdf.
44. Tarasenko, Y.N. and Schoenberg, N.E., *Self-perceived Income sufficiency and self-reported Income level among a health inequity population.* Journal of health care for the poor and underserved, 2017. **28**(2): p. 812-828.

For peer review only

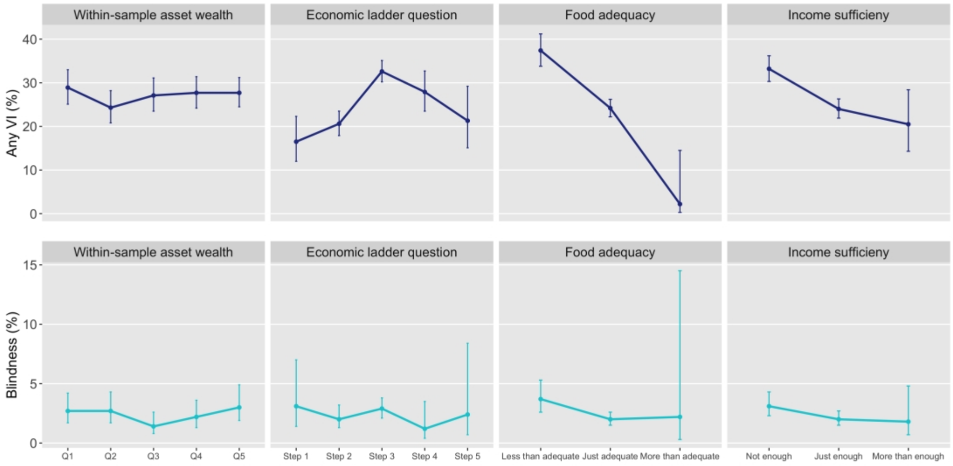

Fig 1. Age-sex weighted prevalence of blindness and any vision impairment in the population 50 years and older by four socioeconomic position variables, The Gambia 2019

1458x749mm (72 x 72 DPI)

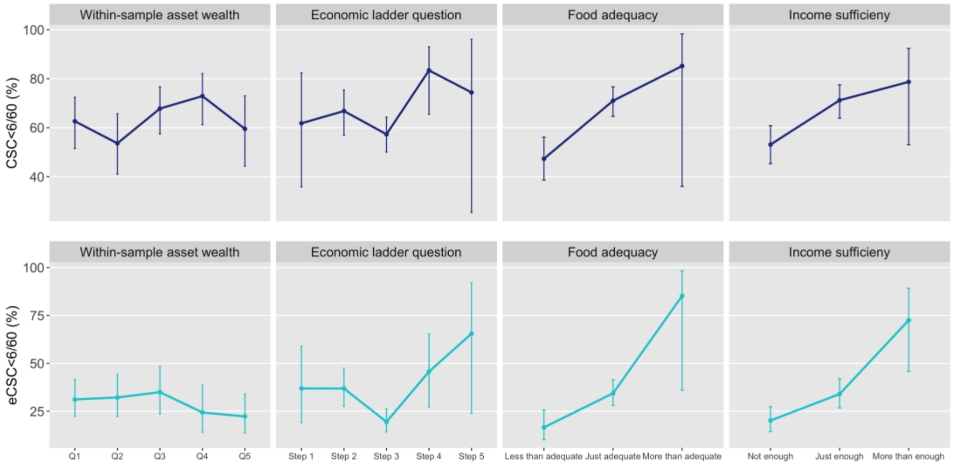

Fig 2. Age-sex weighted cataract surgical coverage and effective cataract surgical coverage (cataract surgical threshold <6/60) in the population 50 years and older by four socioeconomic position variables, The Gambia 2019

1458x749mm (72 x 72 DPI)

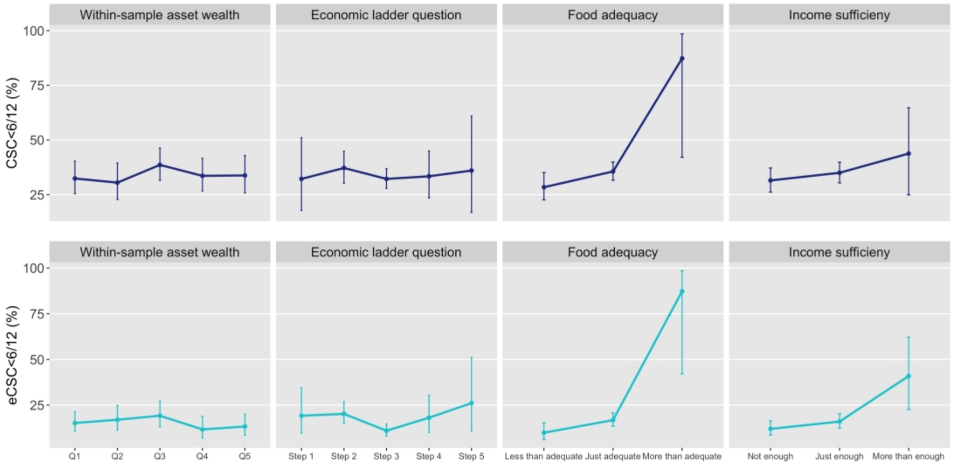

Fig 3. Age-sex weighted cataract surgical coverage and effective cataract surgical coverage (cataract surgical threshold <6/12) in the population 50 years and older by four socioeconomic position variables, The Gambia 2019

1458x749mm (72 x 72 DPI)

Supplementary Material

Table of Contents

1. EQUITYTOOL QUESTIONNAIRE FOR THE GAMBIA ... 1
2. TABLES OF RESULTS USED IN FIGURES 1-3 ... 2

For peer review only

1. EquityTool questionnaire for The Gambia

Released 17 Dec 2018.

Asset list

Does your household have a… (Y/N)

1. Sofa
2. Wardrobe
3. Television
4. Refrigerator
5. DVD/CD Player
6. Satellite Dish
7. Bicycle (belonging to any member of the household)

Household Characteristics

What is the main source of drinking water for members of your household? Specify if other

1. Public tap/ Standpipe
2. Piped to yard/ plot
3. Other water source

What kind of toilet facility do members of your household usually use? Specify if other

1. Flush to septic tank
2. Pit latrine without slab/ Open pit
3. Other toilet type

What is the main material of the floor? Specify if other

1. Ceramic Tile
2. Other floor material

What is the main material of the exterior walls? Specify if other

1. Cement
2. Other

Livestock

How many of the following animals does this household own…

1. Cattle
2. Milk cows or bulls
3. Horses, donkeys or mules
4. Goats
5. Sheep

For peer review only

2. Tables of results used in Figures 1-3

Age-sex weighted prevalence of any vision impairment <6/12 and blindness, and cataract surgical coverage and effective cataract surgical coverage in the population 50 years and older disaggregated by four SEP measures.

Table S1. Weighted prevalence of any vision impairment and blindness and CSC/eCSC across the 2013 DHS EquityTool national scoring within the Gambia 2019 sample aged 50 years and older

	**Quintile 1**	**Quintile 2**	**Quintile 3**	**Quintile 4**	**Quintile 5**
**Population 50+**	N=814	N=794	N=804	N=804	N=804
**Any VI (%)**	28.9 (25.1-33.0)	24.3 (20.8-28.2)	27.1 (23.5-31.1)	27.7 (24.2-31.4)	27.7 (24.5-31.2)
**Blindness (%)**	2.7 (1.7-4.2)	2.7 (1.7-4.3)	1.4 (0.8-2.6)	2.2 (1.3-3.6)	3.0 (1.9-4.9)
**Cataract surgical threshold <6/60**					
**CSC (%)**	62.6 (51.5-72.4)	53.6 (41.0-65.7)	67.8 (57.4-76.7)	72.9 (61.2-82.1)	59.5 (44.3-73.0)
**eCSC (%)**	31.2 (22.3-41.6)	32.2 (22.2-44.2)	35.0 (23.6-48.3)	24.4 (14.1-38.7)	22.3 (13.7-34.1)
**Cataract surgical threshold <6/12**					
**CSC (%)**	32.4 (25.4-40.3)	30.5 (22.8-39.5)	38.6 (31.5-46.3)	33.6 (26.7-41.6)	33.8 (25.8-42.9)
**eCSC (%)**	15.2 (10.6-21.3)	17.0 (11.3-24.8)	19.2 (13.2-27.1)	11.7 (7.0-18.9)	13.3 (8.5-20.1)

Quintiles 1=Poorest, 5=richest

Table S2. Weighted prevalence of any vision impairment and blindness and CSC/eCSC in the bottom 40% and 20% of EquityTool national scoring within the Gambia 2019 sample aged 50 years and older

		**Within-sample ET scoring**			
	**All**	**Top 60%**	**Bottom 40%**	**Top 80%**	**Bottom 20%**
**Population 50+**	N=4020	N=2412	N=1608	N=3206	N=814
**Any VI (%)**	27.2 (25.4-29.0)	27.5 (25.4-29.7)	26.7 (23.8-29.7)	26.7 (24.8-28.7)	28.9 (25.1-33.0)
**Blind (%)**	2.4 (1.9-3.0)	2.2 (1.6-3.0)	2.7 (2.0-3.7)	2.3 (1.8-3.0)	2.7 (1.7-4.2)
**Cataract surgical threshold <6/60**					
**CSC (%)**	63.1 (57.6-68.3)	66.6 (59.2-73.2)	58.4 (50.2-66.2)	63.3 (56.9-69.2)	62.6 (51.5-72.4)
**eCSC (%)**	29.1 (24.2-34.6)	27.2 (20.9-34.6)	31.7 (24.8-39.5)	28.5 (23.0-34.8)	31.2 (22.3-41.6)
**Cataract surgical threshold <6/12**					
**CSC (%)**	33.9 (30.5-37.5)	35.5 (31.1-40.1)	31.6 (26.3-37.4)	34.3 (30.4-38.4)	32.4 (25.4-40.3)
**eCSC (%)**	15.3 (12.6-18.4)	14.8 (11.5-18.9)	16.0 (12.2-20.7)	15.3 (12.3-18.9)	15.2 (10.6-21.3)

Table S3. Weighted prevalence of any vision impairment and blindness and CSC/eCSC across five self-ranked positions on an economic ladder

	**Step 1**	**Step 2**	**Step 3**	**Step 4**	**Step 5**
**Population 50+**	N=227	N=1240	N=1919	N=489	N=112
**Any VI (%)**	16.5 (12.0-22.3)	20.6 (17.9-23.5)	32.6 (30.2-35.1)	27.9 (23.5-32.7)	21.3 (15.1-29.2)
**Blindness (%)**	3.1 (1.4-7.0)	2.0 (1.3-3.2)	2.9 (2.1-3.8)	1.2 (0.4-3.5)	2.4 (0.7-8.4)
**Cataract surgical threshold <6/60**					
**CSC (%)**	61.8 (35.8-82.4)	66.8 (56.9-75.4)	57.3 (50.0-64.3)	83.4 (65.5-93.0)	74.4 (25.4-96.1)
**eCSC (%)**	36.9 (19.2-59.0)	36.9 (27.6-47.4)	19.5 (14.2-26.2)	45.7 (27.3-65.4)	65.5 (23.9-92.0)

For peer review only - http://bmjopen.bmj.com/site/about/guidelines.xhtml

**Cataract surgical threshold <6/12**					
**CSC (%)**	32.2 (17.8-51.0)	37.2 (30.2-44.8)	32.2 (27.9-36.9)	33.4 (23.5-44.9)	36.0 (16.9-60.9)
**eCSC (%)**	19.2 (9.7-34.4)	20.2 (14.9-26.8)	11.0 (8.1-14.7)	18.1 (10.1-30.4)	26.1 (10.7-51.1)

Step 1=Poorest, 5=richest

Table S4. Weighted prevalence of any vision impairment and blindness and CSC/eCSC across three subjective food adequacy levels

	**All**		
	**Less than adequate**	**Just adequate**	**More than adequate**
**Population 50+**	N=976	N=3,006	N=37
**Any VI (%)**	37.4 (33.8-41.2)	24.2 (22.2-26.2)	2.2 (0.3-14.5)
**Blindness (%)**	3.7 (2.6-5.3)	2.0 (1.5-2.6)	2.2 (0.3-14.5)
**Cataract surgical threshold <6/60**			
**CSC (%)**	47.3 (38.6-56.1)	71.0 (64.6-76.7)	85.2 (36.0-98.3)
**eCSC (%)**	16.6 (10.3-25.7)	34.4 (28.1-41.4)	85.2 (36.0-98.3)
**Cataract surgical threshold <6/12**			
**CSC (%)**	28.4 (22.6-35.1)	35.6 (31.6-39.9)	87.3 (42.1-98.5)
**eCSC (%)**	9.9 (6.2-15.3)	16.8 (13.5-20.7)	87.3 (42.1-98.5)

Table S5. Weighted prevalence of any vision impairment and blindness and CSC/eCSC across three subjective income sufficiency levels

	**All**		
	**Less than adequate**	**Just adequate**	**More than adequate**
**Population 50+**	N=1,430	N=2,418	N=172
**Any VI (%)**	33.2 (30.3-36.2)	24.0 (21.9-26.3)	20.5 (14.3-28.4)
**Blindness (%)**	3.1 (2.3-4.3)	2.0 (1.5-2.7)	1.8 (0.7-4.8)
**Cataract surgical threshold <6/60**			
**CSC (%)**	53.1 (45.3-60.8)	71.2 (63.9-77.5)	78.7 (53.0-92.4)
**eCSC (%)**	20.2 (14.4-27.4)	34.0 (26.8-42.0)	72.5 (45.8-89.2)
**Cataract surgical threshold <6/12**			
**CSC (%)**	31.5 (26.2-37.2)	35.0 (30.4-39.8)	43.8 (24.9-64.7)
**eCSC (%)**	12.0 (8.6-16.4)	16.0 (12.4-20.3)	40.9 (22.5-62.2)

For peer review only - http://bmjopen.bmj.com/site/about/guidelines.xhtml

STROBE Statement—Checklist of items that should be included in reports of ***cross-sectional studies***

	Item No	Recommendation	Page No
**Title and abstract**	1	(*a*) Indicate the study's design with a commonly used term in the title or the abstract	1-2
		(*b*) Provide in the abstract an informative and balanced summary of what was done and what was found	2-3
**Introduction**			
Background/rationale	2	Explain the scientific background and rationale for the investigation being reported	4-5
Objectives	3	State specific objectives, including any prespecified hypotheses	5
**Methods**			
Study design	4	Present key elements of study design early in the paper	5
Setting	5	Describe the setting, locations, and relevant dates, including periods of recruitment, exposure, follow-up, and data collection	5-6
Participants	6	(*a*) Give the eligibility criteria, and the sources and methods of selection of participants	5-6
Variables	7	Clearly define all outcomes, exposures, predictors, potential confounders, and effect modifiers. Give diagnostic criteria, if applicable	6-8
Data sources/ measurement	8*	For each variable of interest, give sources of data and details of methods of assessment (measurement). Describe comparability of assessment methods if there is more than one group	6-8
Bias	9	Describe any efforts to address potential sources of bias	9
Study size	10	Explain how the study size was arrived at	5
Quantitative variables	11	Explain how quantitative variables were handled in the analyses. If applicable, describe which groupings were chosen and why	8-9
Statistical methods	12	(*a*) Describe all statistical methods, including those used to control for confounding	8-9
		(*b*) Describe any methods used to examine subgroups and interactions	NA
		(*c*) Explain how missing data were addressed	9
		(*d*) If applicable, describe analytical methods taking account of sampling strategy	9
		(*e*) Describe any sensitivity analyses	NA
**Results**			
Participants	13*	(a) Report numbers of individuals at each stage of study—eg numbers potentially eligible, examined for eligibility, confirmed eligible, included in the study, completing follow-up, and analysed	9
		(b) Give reasons for non-participation at each stage	NA
		(c) Consider use of a flow diagram	NA
Descriptive data	14*	(a) Give characteristics of study participants (eg demographic, clinical, social) and information on exposures and potential confounders	10-11
		(b) Indicate number of participants with missing data for each variable of interest	10-11
Outcome data	15*	Report numbers of outcome events or summary measures	12-14

For peer review only

Main results	16	(*a*) Give unadjusted estimates and, if applicable, confounder-adjusted estimates and their precision (eg, 95% confidence interval). Make clear which confounders were adjusted for and why they were included	12-14
		(*b*) Report category boundaries when continuous variables were categorized	NA
		(*c*) If relevant, consider translating estimates of relative risk into absolute risk for a meaningful time period	NA
Other analyses	17	Report other analyses done—eg analyses of subgroups and interactions, and sensitivity analyses	NA
**Discussion**			
Key results	18	Summarise key results with reference to study objectives	15-18
Limitations	19	Discuss limitations of the study, taking into account sources of potential bias or imprecision. Discuss both direction and magnitude of any potential bias	19-20
Interpretation	20	Give a cautious overall interpretation of results considering objectives, limitations, multiplicity of analyses, results from similar studies, and other relevant evidence	20
Generalisability	21	Discuss the generalisability (external validity) of the study results	20
**Other information**			
Funding	22	Give the source of funding and the role of the funders for the present study and, if applicable, for the original study on which the present article is based	20

*Give information separately for exposed and unexposed groups.

Note: An Explanation and Elaboration article discusses each checklist item and gives methodological background and published examples of transparent reporting. The STROBE checklist is best used in conjunction with this article (freely available on the Web sites of PLoS Medicine at http://www.plosmedicine.org/, Annals of Internal Medicine at http://www.annals.org/, and Epidemiology at http://www.epidem.com/). Information on the STROBE Initiative is available at www.strobe-statement.org.

For peer review only

BMJ Open

Socioeconomic position and eye health outcomes: identifying inequality in rapid population-based surveys

Journal:	*BMJ Open*
Manuscript ID	bmjopen-2022-069325.R1
Article Type:	Original research
Date Submitted by the Author:	19-Jan-2023

Complete List of Authors:	McCormick, Ian; London School of Hygiene & Tropical Medicine, International Centre for Eye Health<br>Kim, Min; London School of Hygiene and Tropical Medicine Faculty of Infectious and Tropical Diseases<br>Hydara, Abba; Sheikh Zayed Regional Eye Care Centre<br>Olaniyan, Segun I; Sheikh Zayed Regional Eye Care Centre<br>Jobe, Modou; MRC Unit The Gambia at LSHTM<br>Badjie, Omar; Ministry of Health, Directorate of Health Promotion and Education<br>Sanyang, Nyakassi MB; Ministry of Finance, Gambia Bureau of Statistics<br>Jarju, Gibril; Ministry of Health, Directorate of Planning and Information<br>Njai, Modou; Ministry of Health, Directorate of Health Promotion and Education<br>Sankareh, Alhagie; Ministry of Health, Regional Directorate of Health Services, West Coast Health Region 1<br>Bastawrous, Andrew; London School of Hygiene & Tropical Medicine, International Centre for Eye Health<br>Allen, Luke; London School of Hygiene & Tropical Medicine, International Centre for Eye Health<br>Mactaggart, Islay; London School of Hygiene and Tropical Medicine, International Centre for Eye Health<br>Burton, Matthew J; London School of Hygiene and Tropical Medicine, International Centre for Eye Health; NIHR Moorfields Biomedical Research Centre<br>Ramke, Jacqueline; London School of Hygiene & Tropical Medicine, International Centre for Eye Health; The University of Auckland, School of Optometry and Vision Science

**Primary Subject Heading**:	Ophthalmology
Secondary Subject Heading:	Epidemiology, Public health, Global health
Keywords:	PUBLIC HEALTH, EPIDEMIOLOGY, OPHTHALMOLOGY

SCHOLARONE™ Manuscripts

I, the Submitting Author has the right to grant and does grant on behalf of all authors of the Work (as defined in the below author licence), an exclusive licence and/or a non-exclusive licence for contributions from authors who are: i) UK Crown employees; ii) where BMJ has agreed a CC-BY licence shall apply, and/or iii) in accordance with the terms applicable for US Federal Government officers or employees acting as part of their official duties; on a worldwide, perpetual, irrevocable, royalty-free basis to BMJ Publishing Group Ltd ("BMJ") its licensees and where the relevant Journal is co-owned by BMJ to the co-owners of the Journal, to publish the Work in this journal and any other BMJ products and to exploit all rights, as set out in our licence.

The Submitting Author accepts and understands that any supply made under these terms is made by BMJ to the Submitting Author unless you are acting as an employee on behalf of your employer or a postgraduate student of an affiliated institution which is paying any applicable article publishing charge ("APC") for Open Access articles. Where the Submitting Author wishes to make the Work available on an Open Access basis (and intends to pay the relevant APC), the terms of reuse of such Open Access shall be governed by a Creative Commons licence – details of these licences and which Creative Commons licence will apply to this Work are set out in our licence referred to above.

Other than as permitted in any relevant BMJ Author's Self Archiving Policies, I confirm this Work has not been accepted for publication elsewhere, is not being considered for publication elsewhere and does not duplicate material already published. I confirm all authors consent to publication of this Work and authorise the granting of this licence.

For peer review only

Title

Socioeconomic position and eye health outcomes: identifying inequality in rapid population-based surveys

Author list

Ian McCormick[1*]
Min J Kim[1]
Abba Hydara[2]
Segun I Olaniyan[2]
Modou Jobe[3]
Omar Badjie[4]
Nyakassi MB Sanyang[5]
Gibril Jarju[6]
Modou Njai[7]
Alhagie Sankareh[8]
Andrew Bastawrous[1]
Luke Allen[1]
Islay Z Mactaggart[1]
Matthew J Burton[1, 9]
Jacqueline Ramke[1, 10]

Affiliations

[1]International Centre for Eye Health, London School of Hygiene & Tropical Medicine, London, UK
[2]Sheikh Zayed Regional Eye Care Centre, Banjul, The Gambia
[3]Medical Research Council Unit The Gambia at London School of Hygiene & Tropical Medicine, Atlantic Boulevard, Fajara, The Gambia
[4]Non-Communicable Disease Programme, Directorate of Health Promotion and Education, Ministry of Health, Kotu, The Gambia
[5]Gambia Bureau of Statistics, Office of the Vice President, Kanifing, The Gambia
[6]Directorate of Planning and Information, Ministry of Health, Kotu, The Gambia
[7]Directorate of Health Promotion and Education, Ministry of Health, Kotu, The Gambia
[8]Regional Directorate of Health Services, Ministry of Health, West Coast Health Region 1, Kanifing, The Gambia
[9]National Institute for Health Research Biomedical Research Centre for Ophthalmology at Moorfields Eye Hospital NHS Foundation Trust and UCL Institute of Ophthalmology, London, UK
[10]School of Optometry and Vision Science, University of Auckland, Auckland, New Zealand

*Corresponding author

Ian McCormick
International Centre for Eye Health, London School of Hygiene & Tropical Medicine, Keppel Street, London, WC1E 7HT, UK
ian.mccormick@lshtm.ac.uk

Word count

4202

For peer review only

ABSTRACT

Objective: Monitoring health outcomes disaggregated by socioeconomic position (SEP) is crucial to ensure no one is left behind in efforts to achieve universal health coverage. In eye health planning, rapid population surveys are most commonly implemented; these need an SEP measure that is feasible to collect within the constraints of a streamlined examination protocol. We aimed to assess whether each of four SEP measures identified inequality – an underserved group or socioeconomic gradient – in key eye health outcomes.

Design: Population-based cross-sectional survey.

Participants: A subset of 4020 adults 50 years and older from a nationally representative sample of 9188 adults aged 35 years and older in The Gambia.

Outcome measures: Blindness (presenting visual acuity [PVA]<3/60), any vision impairment (VI) (PVA<6/12), cataract surgical coverage (CSC) and effective cataract surgical coverage (eCSC) at two operable cataract thresholds (<6/12 and <6/60) analysed by one objective asset-based measure (EquityTool) and three subjective measures of relative SEP (a self-reported economic ladder question and self-reported household food adequacy and income sufficiency).

Results: Subjective household food adequacy and income sufficiency demonstrated a socioeconomic gradient (queuing pattern) in point estimates of any VI and CSC and eCSC at both operable cataract thresholds. Any VI, CSC<6/60 and eCSC<6/60 were worse among people who reported inadequate household food compared to those with just adequate food. Any VI and CSC<6/60 were worse among people who reported not enough household income compared to those with just enough income. Neither the subjective economic ladder question nor the objective asset-wealth measure demonstrated any socioeconomic gradient or pattern of inequality in any of the eye health outcomes.

For peer review only

Conclusion: We recommend pilot-testing self-reported food adequacy and income sufficiency as SEP variables in vision and eye health surveys in other locations, including assessing the acceptability, reliability and repeatability of each question.

STRENGTHS AND LIMITATIONS

- Strengths included the use of a nationally representative sample of adults in a country typical of the low-and-middle income settings surveyed with rapid eye health survey methods
- Further, the outcomes we report are key eye health indicators included in WHO Eye Care Indicator Menu; effective cataract surgical coverage is one of two World Health Assembly endorsed indicators with a global target for 2030
- Also, we report both objective and subjective indicators of socioeconomic position to enable comparison
- Limitations include that the most recent objective asset wealth tool at the time of data collection was based on data collected six years prior (2013 DHS)
- In addition, our sample size was insufficient for analysis of intersectionality of socioeconomic position with other key equity dimensions

For peer review only

For peer review only - http://bmjopen.bmj.com/site/about/guidelines.xhtml

INTRODUCTION

The World Health Organization (WHO) and the *Lancet Global Health* Commission on Global Eye Health have called for vision and eye health data to be disaggregated by equity dimensions.[1, 2] This disaggregation is essential to understand eye health inequalities between population subgroups and to monitor progress towards eye health within Universal Health Coverage. Both WHO and the Commission identified socioeconomic position (SEP) as a key factor in eye health outcomes and the Commission recommended standard SEP indices be included in population-based vision impairment surveys.[1, 2]

Within countries, health inequalities exist along a socioeconomic gradient,[3, 4] and SEP indicators identify people experiencing poverty or deprivation via some means of social stratification.[5-7] SEP indicators can be used to measure the differential consequences of the social determinants of health and to monitor and evaluate policies and programmes designed to address inequity.[8-10] SEP can be described at an individual or group level (e.g., household or community) and can be measured in a variety of ways, such as using income, education, occupation, assets, nutrition, housing or a multidimensional index of such factors.[11] Measures can be objective or subjective, and absolute or relative.[11, 12] Poverty lines and asset wealth are objective measures, while self-reported, subjective measures of SEP assess individuals' perception of their own resources or standing relative to those living around them.

In comprehensive surveys of vision and eye health, lower SEP has been shown to be associated with higher prevalence of vision impairment and worse access to services,[13-16] though there is variation in SEP measures used. The Rapid Assessment of Avoidable Blindness (RAAB) is the most commonly conducted rapid vision survey worldwide.[17, 18] RAAB has streamlined examination protocols compared to comprehensive surveys, but generates key outputs that include the prevalence of vision impairment and effective cataract surgical coverage (eCSC) – both important indicators for monitoring global eye health.[19, 20] Currently, age and gender are the only equity dimensions routinely recorded in

For peer review only - http://bmjopen.bmj.com/site/about/guidelines.xhtml

RAAB while a disability module is optional. To expand data collection to include a measure of SEP within RAAB it is crucial that steps are taken to identify which measures are feasible to collect regularly and might identify those most at risk of being left behind. The anticipated 'time-cost' of incorporating SEP indicators into a rapid survey means more concise questions need to be considered.

In this paper, we aimed to assess whether one objective asset-based measure and three subjective measures of relative SEP demonstrated a socioeconomic gradient or inequality in key eye health outcomes in the population 50 years and older using data from the 2019 Gambia National Eye Health Survey, a comprehensive cross-sectional survey with an embedded RAAB.[21, 22] Further, we aimed to assess the level of association between the objective asset based measure and three subjective measures of relative SEP and recommend one or more indicators to pilot test in other locations.

METHODS

Data collection and categorisation

A detailed summary of the 2019 Gambia National Eye Health Survey sampling and examination protocol is provided elsewhere.[21] Briefly, the 2013 National Census was used as a sampling frame to select a nationally representative sample of adults aged 35 years and older in clusters of 30 people.[23] A multi-stage, stratified, cluster random sampling with probability proportionate to size strategy was used. The sample was stratified by historic geographic regions of the country and the urban and rural population proportions within them, using The Gambia Bureau of Statistics' definitions of urban and rural.

At the individual level, sociodemographic data collected included age, sex, marital status, religion, ethnicity, education, occupation and subjective socioeconomic position (details below). A questionnaire on asset ownership was completed at the household level via the household head or an adult household key informant (details below). No absolute measure of income or expenditure was collected. Distance visual acuity (VA) was measured indoors at 3 metres using the Peek Acuity

For peer review only

application on Huawei MediaPad M3 tablet devices;[24] more information is provided in the Eye Health Assessment section of the study protocol.[21] Lens grading was done on slit-lamp examination following dilation with tropicamide 1% eye drops and according to the WHO Cataract Grading Tool.[25] The survey was completed by four data collection teams between February and July 2019.

Eye health outcomes

We selected WHO priority national eye health indicators as outcomes: vision impairment prevalence and effective cataract surgical coverage.[26] We report blindness and vision impairment according to existing WHO definitions. Cataract surgical coverage (CSC) and effective cataract surgical coverage (eCSC) were calculated using the most recent definition.[27] For the purposes of determining service coverage, cataract classified as gradable, mature or hypermature met the definition of *operable*. *Operated* cataract was defined where aphakia (excluding cases of couching) or an intraocular lens was recorded. CSC and eCSC were reported at two best-corrected VA surgical thresholds: <6/12 and <6/60, and the threshold for effective coverage (post-operative presenting VA) was ≥6/12.[19]

Box 1. Four key eye health outcomes and their descriptions.

**Eye health outcome**	**Description**
Blindness	Presenting VA (i.e., with available correction, if worn) in the better eye worse than 3/60
Any vision impairment (VI)	Presenting VA (i.e., with available correction, if worn) in the better eye worse than 6/12
Cataract surgical coverage (CSC)	The number of people with operated cataract as a proportion of those having operable plus operated cataract
Effective cataract surgical coverage (eCSC)	The number of people with operated cataract and a good visual outcome (presenting VA 6/12 or better) as a proportion of those having operable plus operated cataract

For peer review only - http://bmjopen.bmj.com/site/about/guidelines.xhtml

SEP indicators

SEP was assessed in four separate ways. The first was to divide the study population into SEP quintiles using the 'EquityTool' analysis provided by Metrics for Management (www.m4mgmt.org). EquityTool is an *objective relative SEP measure* that uses a short asset ownership and household characteristics questionnaire to generate wealth quintiles with a minimum level of agreement (Kappa ≥0.75) with quintiles derived from underlying (more comprehensive) Demographic and Health Survey (DHS) data. The EquityTool for The Gambia used the 2013 DHS and consisted of 16 questions (appendix p 1).[28] Participants were assigned to a national wealth quintile benchmarked to the 2013 DHS according to their household questionnaire score. In addition to quintile assignment, the EquityTool output includes three continuous variables for national, urban and rural scoring on the questionnaire. We created evenly distributed within-sample quintiles based on the national score for all participants. These were not benchmarked to national thresholds and represented the distribution of asset wealth within the sample only.

For the remaining three questions we used *subjective relative SEP measures* relating to food adequacy, income sufficiency and self-perceived position on an economic ladder (Box 2). These questions come from Howe and colleagues' analysis of 11,280 Malawian households that found them to be better at identifying low-income households (those living on less than US$1/ day) than a composite wealth index.[8]

Box 2. Three subjective socioeconomic position variables including the question and possible responses.

Food adequacy	"When you think about the food in your household would you say you have 1) Less than adequate food for the needs of your household, 2) Just adequate food for the needs of your household or 3) More than adequate food for the needs of your household."

For peer review only

Income sufficiency	"When you think about the income in your household would you say it is 1) Not enough to cover our needs, we must borrow, 2) Not enough to cover our needs, we use savings, 3) Just enough to cover our needs, 4) Enough to cover our needs, we are able to save a little or 5) Enough to cover our needs, we are building savings."
Economic ladder question	"Please look at this ladder [5 step ladder pictured]. If the bottom step (1) of the ladder represents the people who are poorest in your community, and the top step (5) represents the people who are richest, on which step of the ladder do you feel your household stands on?"

Patterns of inequality

We sought to identify which of the patterns of inequality described in the WHO *Handbook on Health Inequality Monitoring*, were present for our outcomes, namely, mass deprivation, marginal exclusion, queuing or complete coverage.[9] Mass deprivation describes poor health outcomes in all SEP groups except the richest. Marginal exclusion describes worse outcomes in the poorest group compared to all other. Queuing describes an approximately linear decrease in outcomes down SEP groups. Complete coverage describes universally good outcomes across SEP groups.

Data analysis

In reporting selected eye health outcomes, we restricted our population of interest to people 50 years and older as this age group is directly relevant to the RAAB survey methodology. We assessed the association between the ordinal categories of objective relative asset wealth and each subjective SEP measure by estimating Kendall's tau-b correlation coefficient (assuming a monotonic relationship for all three comparisons). Kendall's tau-b coefficient values range from -1 to 1, with values closer to 1 indicating a stronger relationship and a negative value indicating an inverse relationship.

For peer review only - http://bmjopen.bmj.com/site/about/guidelines.xhtml

All analyses were done in Stata 16. We assigned participants aged 50 years and older to within-sample wealth quintiles using the xtile command. The prevalence of any VI, blindness, CSC and eCSC and their 95% confidence intervals were calculated adjusted for clustering and post-stratified (weighted) according to the age-sex distribution of The Gambia 2013 Census using the svy command. The weighted proportions of participants by sociodemographic and socioeconomic position characteristics were calculated according to the age-sex distribution of The Gambia 2013 Census using the svy command.

For peer review only

Missing data

To prevent listwise deletion during analyses, raw data were checked for completeness. In August 2020, follow-up data collection was completed by telephone to address some missing EquityTool data and following this, 600 forms with persisting missing critical data (one or more entire module [e.g., optometry, ophthalmology, mental health], EquityTool data or visual acuity data) were deleted from the final sample.[21] EquityTool questions were imputed for 398 participants (4.3%) in the final dataset (179 [4.5%] participants aged 50 years and older), whereby missing values in a cluster were replaced with the most frequent, non-missing value from within the cluster. Missing values were not substituted for questions that had more than one "most frequent" response in the cluster (e.g., bimodal with equal numbers of observed values for "yes" and "no").[21]

Patient and public involvement

Survey participants were not involved in the design of the survey.

RESULTS

There were 9,188 participants examined out of 11,027 enumerated (response rate 83%). The mean age of the sample was 49.6 years (standard deviation 13.4), 71% were female and 55% were living in an urban location. There were 4020 participants aged 50 years or older included in the sample. The

For peer review only - http://bmjopen.bmj.com/site/about/guidelines.xhtml

sociodemographic and socioeconomic characteristics of the subgroup aged 50 years and older are presented in Table 1.

Table 1. Sociodemographic and socioeconomic position characteristics in the population 50 years and older, The Gambia National Eye Health Survey, 2019.

	Population 50 years and older (N=4020)		
	N	Crude %	Weighted %
**Sex**			
Male	1,576	39.2	50.9
Female	2,444	60.8	49.1
**Marital status**			
Married/ cohabiting	2,912	72.4	76.3
Widowed	1,035	25.8	21.8
Divorced/ separated	57	1.4	1.4
Never married	16	0.4	0.5
**Location**			
Urban	2,180	54.2	54.8
Rural	1,840	45.8	45.2
**Education level**			
Non-formal (Quranic)[a]	2,455	61.1	58.9
Pre-school[b]/ no school	823	20.5	20.4
Primary[b]	229	5.7	6.1
Secondary[b]/ vocational	318	7.9	9.5
Higher	86	2.1	2.7
Don't know	109	2.7	2.4
**Occupation category**			
Unemployed	910	22.6	21.4
Manual/ unskilled	1,987	49.4	47.8
Trade/ skilled manual	714	17.8	19.4
Professional	120	3.0	3.9

For peer review only - http://bmjopen.bmj.com/site/about/guidelines.xhtml

Retired	238	5.9	5.9
Other	51	1.3	1.6
**National asset wealth quintiles (2013 DHS EquityTool)**			
Q1 - poorest	366	9.1	9.4
Q2	547	13.6	14.1
Q3	1,022	25.4	25.3
Q4	936	23.3	24.0
Q5 - richest	1,149	28.6	27.3
**Within-sample asset wealth quintiles**			
Q1 - poorest	814	20.3	-
Q2	794	19.8	-
Q3	804	20.0	-
Q4	804	20.0	-
Q5 - richest	804	20.0	-
**Food adequacy**			
Less than adequate	976	24.3	24.3
Just adequate	3,006	74.8	74.7
More than adequate	37	0.9	1.0
Missing	1	0.0	-
**Income sufficiency**			
Not enough, must borrow	922	22.9	22.6
Not enough, use savings	508	12.6	13.2
Just enough	2,418	60.2	59.9
Enough, save a little	167	4.2	4.1
Enough, build savings	5	0.1	0.1
Missing	0	0.0	-
**Economic ladder question (ELQ)**			
Step 1 - poorest	227	5.7	5.5
Step 2	1,240	30.9	29.5
Step 3	1,919	47.7	49.3
Step 4	489	12.2	12.8

For peer review only - http://bmjopen.bmj.com/site/about/guidelines.xhtml

Step 5 - richest	112	2.8	3.0
Missing	33	0.8	-

[a]Non-formal (Quranic) describes a system where students study mostly religious education at home for a non-specific number of years without any formal curriculum; [b]Includes Madrassa and non-Madrassa systems (Madrassa describes schools where Arabic is the medium of instruction and with emphasis on Islamic education)

There was an uneven distribution of survey participants across the national EquityTool quintiles generated from the 2013 DHS. The poorest two quintiles were under-represented (only 9% of the sample were in the poorest quintile [Q1] and 14% were in Q2) while the wealthiest three quintiles were over-represented (Table 1). For all subsequent analyses, in place of these groups, we used the evenly distributed within-sample asset wealth quintiles that we generated. Very few participants placed themselves in the wealthiest category of any of the three subjective measures. For subsequent analyses we combined the two highest categories of income sufficiency because of the very small number of participants in the wealthiest group and combined the poorest two groups to leave three levels, consistent with the food adequacy measure. Over half the participants had received a non-formal (Quranic) education; this did not allow for an ordinal scale of education levels to be compared with other SEP measures and education was not included in the analyses.

Relationship between objective and subjective SEP measures in the sample 50 years and older

For the population 50 years and older, the within-sample asset wealth quintiles showed weak positive correlation with each of the subjective food adequacy, income sufficiency and economic ladder question responses (Kendall's tau-b statistic (τ_b) = 0.09, 0.15 and 0.18 respectively; N=4019, 4020 and 3987 respectively; p<0.001 for all three estimates).

Key eye health outcomes among the population 50 years and older

Among the total population 50 years and older, the age-sex weighted prevalence of blindness was 2.4% (95% confidence interval [CI] 1.9-3.0%) and of any VI was 27.2% (95% CI 25.4-29.0%). In the same population, the age-sex weighted CSC and eCSC were 63.1% (95% CI 57.6-68.3%) and 29.1% (24.2-

34.6%) respectively at the <6/60 threshold for cataract surgery. At the lower threshold for cataract surgery of <6/12, the CSC and eCSC estimates were 33.9% (30.5-37.5%) and 15.3% (12.6-18.4%) respectively.

Weighted prevalence of vision impairment and cataract surgical coverage in the population 50 years and older disaggregated by SEP measures

Objective relative asset wealth (EquityTool)

No pattern of inequality was identified across within-sample wealth quintiles for any VI, blindness, CSC or eCSC at either threshold and there were no statistically significant differences between any of the quintiles (Figures 1-3 and appendix Table S1). Consolidating within-sample quintiles to a binary comparison of asset wealth groups showed the weighted prevalence of any VI and blindness and weighted coverage (by any measure) in the poorest one or two quintiles was not significantly different to the top four or three respectively, or to the overall weighted prevalence or coverage estimates (appendix Table S2).

Fig 1. Age-sex weighted prevalence of blindness and any vision impairment in the population 50 years and older by four socioeconomic position variables, The Gambia 2019

Fig 2. Age-sex weighted cataract surgical coverage and effective cataract surgical coverage (cataract surgical threshold <6/60) in the population 50 years and older by four socioeconomic position variables, The Gambia 2019

Fig 3. Age-sex weighted cataract surgical coverage and effective cataract surgical coverage (cataract surgical threshold <6/12) in the population 50 years and older by four socioeconomic position variables, The Gambia 2019

Subjective measure: Economic Ladder Question

Almost half of participants placed themselves on the middle position of the ladder (step 3); three quarters of responses were ‘clumped’ together across steps 2 and 3 (Table 1). There was no pattern

For peer review only - http://bmjopen.bmj.com/site/about/guidelines.xhtml

of inequality in any VI, blindness, CSC or eCSC across the five economic ladder steps. Step 3 (the largest group) had a significantly higher prevalence of any VI compared to steps 1, 2 and 5 while there were no significant differences in blindness prevalence across the ladder (Figure 1). There were few participants or cataract cases placed at either end of the scale and confidence intervals for the estimates in these positions were wide, however, $eCSC_{<6/60}$ was significantly higher for people who placed themselves on step 2 compared to step 3 (Figure 2). See appendix Table S3.

Subjective measure: Household food adequacy

Only 1% of people reported having more than adequate household food, while 24% reported less than adequate household food (Table 1). Considering the point estimates, a queuing pattern of inequality was apparent across food adequacy levels for almost all eye health outcomes, however, confidence intervals for the estimates in the top group were very wide. Any VI, blindness, $CSC_{<6/60}$ and $eCSC_{<6/60}$ were all significantly worse among those with less than adequate food compared to those with just adequate food (Figures 1 & 2) while the difference between the two was not significant for $CSC_{<6/12}$ and $eCSC_{<6/12}$ (Figure 3). See appendix Table S4.

Subjective measure: Household income sufficiency

After consolidating the original five levels of income sufficiency to three, still only 4% of people reported 'more than enough' household income to be able to 'save a little' (level 4) or 'build savings' (level 5). Combined, 36% of participants occupied the two original 'not enough' levels of household income ('must borrow' and 'use savings'). See appendix Table S5. Considering the point estimates, a queuing pattern of inequality was seen across three levels of income sufficiency for all eye health outcomes. As for food adequacy, the low numbers of participants and cataract cases in the top group meant confidence intervals were very wide, but there were significant differences in any VI and $CSC_{<6/60}$ between the middle and bottom groups (Figures 1 & 2).

For peer review only - http://bmjopen.bmj.com/site/about/guidelines.xhtml

DISCUSSION

The 2019 Gambia National Eye Health Survey estimated key eye health outcomes in a nationally representative sample of adults aged 35 years and older and included one objective measure of asset-based relative wealth (EquityTool) and three subjective measures of socioeconomic position. We analysed data on a sub-group of 4020 adults aged 50 years and older.

The Gambia's 2015-16 Integrated Household Survey estimated an absolute measure of poverty (poverty line) based on per capita consumption. The proportion of the population living in poverty was 48.6%, an increase of 0.5% since 2010.[29] While an absolute measure like this may be a more intuitive concept of poverty, we focussed on relative SEP measures as they identify inequality in all settings (including where absolute poverty decreases with development) and are well aligned with the aim of monitoring eye health within UHC.

We found no patterns of inequality or significant differences between quintiles for any of our selected eye health outcomes by relative objective asset wealth. Similarly, a 2020 RAAB survey in rural Nigeria included an EquityTool questionnaire benchmarked to 2013 national DHS data.[30] The proportion of participants in each quintile was not reported, but there was no pattern of inequality or significant differences in blindness, severe VI or moderate VI across wealth quintiles. Asset-based measures such as EquityTool are easier to capture than income or expenditure, particularly in LMIC, and participant responses can often be verified at interview.[8] However, certain assets may have a different relationship with SEP in different subgroups, which must be accounted for in analyses and interpretation. Even then, asset ownership does not distinguish between asset quality and assets may not reflect the short-term resource availability often required to make out-of-pocket payments for health care.[31] In addition, wealth indices may conflate community-level factors with household-level factors,[8] and where national thresholds are used in a relatively homogenous sub-national region, as can be surveyed with RAAB, respondents may be 'clumped' in one or two quintiles.[32] From the perspective of a rapid survey methodology, multiple additional questions per individual or household

For peer review only

may increase the time and cost of data collection to the point where it becomes a barrier to eye health planners embarking on a survey.

Howe et al. proposed three desirable features of an SEP indicator for use in health research or evaluation in LMIC: that it is feasible in terms of cost and complexity, reliable and reproducible and that the underlying social stratification process be understood.[8] In terms of their simplicity, any of the three subjective SEP measures reviewed here would be feasible to incorporate into a rapid survey protocol and the stratification generated by each is straightforward.

The subjective economic ladder question did not identify any pattern of inequality in outcomes across the five steps. In the two most frequently selected positions (steps 2 and 3), any VI and $eCSC_{<6/60}$ were significantly better in the poorer of the two SEP positions. However, subjective socioeconomic position has been shown to be a useful predictor of health in older people elsewhere,[33] and the economic ladder question may be worth reassessing in vision and eye health surveys in other settings. We presented an image of a ladder with five steps. Participants may have been distributed differently across a nine- or ten-step ladder (as has been used in other studies[34, 35]) which may have shown a pattern of inequality not identified here.

The three-tiered subjective measures of household food adequacy and income sufficiency demonstrated that very few people in The Gambia self-identify as having more than enough of either resource. The concept of food security encompasses the need to have the physical or financial means to obtain quality food on a regular and sustained basis.[36] In LMIC, food has been shown to be households' single biggest expense,[37] and food inadequacy may be a useful tool to identify the people with fewest resources in such settings. In 2015-16, food accounted for almost 60% of total household consumption expenditure in The Gambia.[29] A direct, subjective measure of food adequacy may be better than objective measures of adequacy, such as consumption or body mass index, given natural variations in physiology and the absence of agreed norms.[38] Historically, The Gambia has suffered from food insecurity, with levels increasing in recent years on account of climate-related flood and

For peer review only - http://bmjopen.bmj.com/site/about/guidelines.xhtml

drought.[39] Almost a quarter of participants aged 50 years and older reported less than adequate household food and people who self-identified in this bottom group experienced worse eye health outcomes than those in the middle group. Outcomes were best in the top group but confidence intervals for the estimates were very wide due to the very small group size. Food poverty is also prevalent in high-income countries and also associated with households' financial resources.[36] In high and some middle income settings, the smallest group may commonly be the 'less than adequate' group rather than the 'more than adequate' seen here. In these settings, a binary comparison can be made between 'more than adequate' and 'just adequate' in place of 'just adequate' and 'less than adequate'.

Subjective income sufficiency has a complex relationship with actual income and subjective well-being and may vary over the life course.[40] In a study of people 50 years and older across twelve high-income countries it was most strongly associated with individuals' net worth and employment status, but may be over-estimated in the oldest age groups.[40] In The Gambia, just over a third of participants aged 50 years and older reported insufficient household income such that the household had to borrow money or spend savings to meet their needs. Eye health outcomes stratified by self-reported household income sufficiency followed a similar trend to food adequacy. Point estimates were all lowest in the bottom group, with any VI and $CSC_{<6/60}$ outcomes significantly worse than the middle group.

The distribution of responses to food adequacy and income sufficiency limited the extent to which any pattern of inequality could be reliably interpreted as the measures were effectively reduced to binary "not enough" vs "just enough" comparisons. However, as potential eye health equity indicators, they were able to demonstrate that people who perceived themselves to have the least resources were significantly worse-off for any VI prevalence, and CSC and eCSC at the <6/60 threshold. In The Gambia, eCSC was low in the overall population 50 years and older at both thresholds ($eCSC_{<6/60}$ 30%; $eCSC_{<6/12}$ 15%). Point estimates for those with insufficient income were lower (21% and 12% respectively) and lower again for inadequate food (16% and 10% respectively). These subjective, experience-based SEP

For peer review only - http://bmjopen.bmj.com/site/about/guidelines.xhtml

measures may capture less obvious aspects of social status and allow research participants to be centred in the process of identifying socioeconomic inequality in each survey setting.[8] Tracking eCSC in socioeconomic groups stratified by food adequacy and/ or income sufficiency may be a useful way to monitor eye health equity within countries over time and is aligned with WHO's emphasis on countries achieving a 30 percentage points increase in all population sub-groups. Unlike SEP quintiles, the relative size of the self-reported groups may increase or decrease over time and, alongside changing effective coverage, this information may also be useful for national and sub-national service planning.

The objective relative asset wealth metric showed weak positive association with each of the three subjective SEP measures used. Varying levels of agreement between objective and subjective SEP metrics have been reported in the literature. In eye health, a cataract vision impairment and poverty case-control study across three low- and middle-income countries found that measures of per capita expenditure, asset ownership and self-rated wealth were highly correlated.[37] A trachoma and poverty case-control study in Ethiopia found moderate agreement between a within-study asset-wealth index and self-rated wealth.[41] A 2004-2005 national household survey in Malawi found poor agreement between an objective asset-wealth index and the same subjective SEP measures we used here and the authors noted that the two approaches appeared to be measuring different concepts.[8] Individual indicators of SEP may be correlated but are rarely proxies for each other as they variously represent unique and shared aspects of the concept.[42] However, to identify a socioeconomic group at risk of – or demonstrate a socioeconomic gradient in – a poor health outcome such as vision impairment, the choice of relative SEP indicator may not be crucial.[8]

RAAB surveys can be nationally representative but have more often been carried out at the subnational level, where participants' place of residence is often either predominantly rural or urban. We considered our sample size of around 4000 people 50 years and older too small to examine the effect of intersectionality on eye health outcomes and did not report outcomes by SEP in urban and

For peer review only - http://bmjopen.bmj.com/site/about/guidelines.xhtml

rural populations separately. This is of relevance for rare outcomes such as blindness and cataract service coverage indicators where the number of observations in subgroups may be small. However, intersectionality has been shown to be an important consideration in eye health,[43] and disaggregation by two equity dimensions may be feasible for some outcomes in larger RAAB surveys. Further research is required to address this question in future surveys. Another consideration for future research is the use of small-area deprivation indices such as those already employed in some high-income settings.[44] Pre-existing metrics could be applied at the cluster level and be used alongside or instead of individual level SEP data.

Our study had several limitations. Our sample was not evenly distributed across the quintiles of asset wealth generated by the EquityTool based on the 2013 DHS. Another DHS was completed in The Gambia in 2019-2020 but the subsequent EquityTool was unavailable at the time of our survey. The questions in the EquityTool elicit durable asset ownership and household characteristics. Given six years had passed between the 2013 DHS on which the Equity Tool was based and our survey, we assume ongoing urbanisation and improved water and sanitation likely contributed to the under-representation of lower SEP quintiles and over-representation of higher SEP quintiles in our sample.[45] There may have been stronger associations between participant responses to the subjective SEP questions and a more contemporary or comprehensive asset wealth index. We did not assess the reliability or repeatability of the subjective SEP measures in this study, nor did we assess their acceptability in the population; however, previous research in a low-income population in the USA indicates self-reporting subjective income sufficiency may be less contentious than reporting a specific income level.[46] The wording of the questions may need to be refined in different settings to ensure they make sense to the population. These issues should all be addressed in future research. A comparison of the effects of different SEP dimensions (including those not described here) on VI status may help identify which specific socioeconomic characteristics or resources are most important for good eye health outcomes. We did not try to estimate the effects of various SEP measures on e.g.,

For peer review only

For peer review only - http://bmjopen.bmj.com/site/about/guidelines.xhtml

vision impairment prevalence. Any such analysis of multiple SEP indicators requires careful causal interpretation to avoid mutual adjustment fallacies, and was beyond the aims of this study.[42]

We propose pilot testing the inclusion of two subjective SEP indicators – food adequacy and income sufficiency – in future RAAB surveys. Both SEP indicators demonstrated an inequality gradient in eye health outcomes in the population 50 years and older and may be useful for tracking progress in eCSC among population groups most in need of services. These indicators should be tested in a variety of countries with different income levels and the acceptability of each question should be reviewed in different cultural contexts.

COMPETING INTERESTS

None declared.

FUNDING

This research was funded by The Queen Elizabeth Diamond Jubilee Trust [Grant number TG002]. MJB is supported by the Wellcome Trust [207472/Z/17/Z].

AUTHOR CONTRIBUTIONS

JR conceptualised the research question. IM, MJK, AH, SIO, MJ, AB, IZM, MJB and JR contributed to the design and conduct of the survey. IM, MJK and IZM analysed the data. OB, NMBS, GJ, MN, AS and LA contributed to interpreting the results. IM drafted the original manuscript. All co-authors reviewed and edited the manuscript.

ACKNOWLEDGEMENT

JR's position at the University of Auckland is funded by the Buchanan Charitable Foundation, New Zealand.

For peer review only - http://bmjopen.bmj.com/site/about/guidelines.xhtml

ETHICS APPROVAL

Ethical approval for the study was granted by The Gambia Government/Medical Research Council Joint Ethics and Scientific Coordinating Committee (Ref SCC 1635) and the London School of Hygiene & Tropical Medicine Research Ethics Committee (Ref 16172). All participants provided written informed consent to participate in the 2019 Gambia National Eye Health Survey.

DATA SHARING STATEMENT

The anonymised dataset will be made available on reasonable request from the study team.

For peer review only

For peer review only - http://bmjopen.bmj.com/site/about/guidelines.xhtml

References

1. World Health Organization, *World report on vision*. 2019: Geneva.
2. Burton, M.J., Ramke, J., Marques, A.P., et al., *The Lancet Global Health Commission on Global Eye Health: vision beyond 2020.* The Lancet Global Health, 2021. **9**(4): p. e489-e551.
3. Marmot, M., *Social determinants of health inequalities.* The Lancet, 2005. **365**(9464): p. 1099-1104.
4. Marmot, M., Friel, S., Bell, R., Houweling, T.A.J., and Taylor, S., *Closing the gap in a generation: health equity through action on the social determinants of health.* The Lancet, 2008. **372**(9650): p. 1661-1669.
5. Galobardes, B., Shaw, M., Lawlor, D.A., Lynch, J.W., and Davey Smith, G., *Indicators of socioeconomic position (part 1).* Journal of Epidemiology and Community Health, 2006. **60**(1): p. 7.
6. Galobardes, B., Shaw, M., Lawlor, D.A., Lynch, J.W., and Davey Smith, G., *Indicators of socioeconomic position (part 2).* Journal of epidemiology and community health, 2006. **60**(2): p. 95-101.
7. Galobardes, B., Lynch, J., and Smith, G.D., *Measuring socioeconomic position in health research.* British Medical Bulletin, 2007. **81-82**(1): p. 21-37.
8. Howe, L.D., Hargreaves, J.R., Ploubidis, G.B., De Stavola, B.L., and Huttly, S.R., *Subjective measures of socio-economic position and the wealth index: a comparative analysis.* Health Policy Plan, 2011. **26**(3): p. 223-32.
9. World Health Organization, *Handbook on health inequality monitoring: with a special focus on low-and middle-income countries*. 2013: World Health Organization.
10. World Health Organization, *A conceptual framework for action on the social determinants of health.* 2010.
11. Krieger, N., Williams, D.R., and Moss, N.E., *Measuring social class in US public health research: concepts, methodologies, and guidelines.* Annu Rev Public Health, 1997. **18**: p. 341-78.
12. Conway, D.I., McMahon, A.D., Brown, D., and Leyland, A.H., *Measuring socioeconomic status and inequalities*, in *Reducing social inequalities in cancer: evidence and priorities for research*. 2019: Lyon, France.
13. Gilbert, C.E., Shah, S.P., Jadoon, M.Z., et al., *Poverty and blindness in Pakistan: results from the Pakistan national blindness and visual impairment survey.* BMJ (Clinical research ed.), 2008. **336**(7634): p. 29-32.
14. Tafida, A., Kyari, F., Abdull, M.M., et al., *Poverty and Blindness in Nigeria: Results from the National Survey of Blindness and Visual Impairment.* Ophthalmic Epidemiology, 2015. **22**(5): p. 333-341.
15. Bourne, R.R., Dineen, B.P., Huq, D.M., Ali, S.M., and Johnson, G.J., *Correction of refractive error in the adult population of Bangladesh: meeting the unmet need.* Invest Ophthalmol Vis Sci, 2004. **45**(2): p. 410-7.
16. Braithwaite, T., Verlander, N.Q., Peto, T., et al., *National Eye Survey of Trinidad and Tobago (NESTT): prevalence, causes and risk factors for presenting vision impairment in adults over 40 years.* British Journal of Ophthalmology, 2020. **104**(1): p. 74-80.
17. Mactaggart, I., Limburg, H., Bastawrous, A., Burton, M.J., and Kuper, H., *Rapid Assessment of Avoidable Blindness: looking back, looking forward.* The British journal of ophthalmology, 2019. **103**(11): p. 1549-1552.
18. Mactaggart, I., Wallace, S., Ramke, J., et al., *Rapid assessment of avoidable blindness for health service planning.* Bulletin of the World Health Organization, 2018. **96**(10): p. 726.
19. Keel, S., Müller, A., Block, S., et al., *Keeping an eye on eye care: monitoring progress towards effective coverage.* The Lancet Global Health, 2021.

For peer review only - http://bmjopen.bmj.com/site/about/guidelines.xhtml

20. McCormick, I., Mactaggart, I., Resnikoff, S., et al., *Eye health indicators for universal health coverage: results of a global expert prioritisation process.* British Journal of Ophthalmology, 2021: p. bjophthalmol-2020-318481.
21. Hydara, A., Bastawrous, A., Bell, S., et al., *The Gambia National Eye Health Survey 2019: survey protocol [version 2; peer review: 2 approved].* Wellcome Open Research, 2021. **6**(10).
22. Hydara, A., Mactaggart, I., Bell, S.J., et al., *Prevalence of blindness and distance vision impairment in the Gambia across three decades of eye health programming.* British Journal of Ophthalmology, 2021: p. bjophthalmol-2021-320008.
23. Gambia Bureau of Statistics, *The Gambia 2013 Population and Housing Census Preliminary Results*. 2013, Gambia Bureau of Statistics: Serrekunda, The Gambia.
24. Bastawrous, A., Rono, H.K., Livingstone, I.A., et al., *Development and Validation of a Smartphone-Based Visual Acuity Test (Peek Acuity) for Clinical Practice and Community-Based Fieldwork.* JAMA Ophthalmol, 2015. **133**(8): p. 930-7.
25. Thylefors, B., Chylack, L.T., Jr., Konyama, K., et al., *A simplified cataract grading system.* Ophthalmic Epidemiol, 2002. **9**(2): p. 83-95.
26. World Health Organization, *Eye care indicator menu (ECIM): a tool for monitoring strategies and actions for eye care provision*. 2022, Geneva: World Health Organization.
27. McCormick, I., Butcher, R., Evans, J.R., et al., *Effective cataract surgical coverage in adults aged 50 years and older: estimates from population-based surveys in 55 countries.* The Lancet Global Health, 2022.
28. Metrics for Management. *Gambia EquityTool*. 2018; Available from: equitytool.org.
29. Gambia Bureau of Statistics. *Integrated Household Survey 2015/16: Vol III Prevalence and depth of poverty*. 2017 [cited 2021 12.10]; Available from: https://www.gbosdata.org/downloads/integrated-household-survey-ihs-24.
30. Gascoyne, B., Jolley, E., Ogundimu, K., Chailloux, A., and Owoeye, J. *Rapid Assessment of Avoidable Blindness (RAAB) report, Kogi State, Nigeria*. 2020 [cited 2021 19.10]; Available from: https://research.sightsavers.org/wp-content/uploads/sites/8/2021/03/Rapid-Assessment-of-Avoidable-Blindness-RAAB-report-Kogi-State-Nigeria-IF-October-2020.pdf.
31. Vyas, S. and Kumaranayake, L., *Constructing socio-economic status indices: how to use principal components analysis.* Health Policy Plan, 2006. **21**(6): p. 459-68.
32. Jolley, E., Buttan, S., Engels, T., et al., *Prevalence of Visual Impairment and Coverage of Cataract Surgical Services: Associations with Sex, Disability, and Economic Status in Five Diverse Sites.* Ophthalmic Epidemiology, 2020. **27**(6): p. 429-437.
33. Demakakos, P., Nazroo, J., Breeze, E., and Marmot, M., *Socioeconomic status and health: The role of subjective social status.* Social Science & Medicine, 2008. **67**(2): p. 330-340.
34. Adler, N.E., Epel, E.S., Castellazzo, G., and Ickovics, J.R., *Relationship of subjective and objective social status with psychological and physiological functioning: Preliminary data in healthy, White women.* Health psychology, 2000. **19**(6): p. 586.
35. Ravallion, M. and Lokshin, M., *Subjective economic welfare*. Vol. 2106. 1999: World Bank Publications.
36. Gorton, D., Bullen, C.R., and Mhurchu, C.N., *Environmental influences on food security in high-income countries.* Nutrition Reviews, 2010. **68**(1): p. 1-29.
37. Kuper, H., Polack, S., Eusebio, C., et al., *A case-control study to assess the relationship between poverty and visual impairment from cataract in Kenya, the Philippines, and Bangladesh.* PLoS Med, 2008. **5**(12): p. e244.
38. Mahadevan, R. and Hoang, V., *Is There a Link Between Poverty and Food Security?* Social Indicators Research, 2016. **128**(1): p. 179-199.
39. World Food Programme. *Gambia Country Profile*. 2021 [cited 2021 23.06]; Available from: https://www.wfp.org/countries/gambia.

For peer review only

40. Litwin, H. and Sapir, E.V., *Perceived income adequacy among older adults in 12 countries: findings from the survey of health, ageing, and retirement in Europe.* Gerontologist, 2009. **49**(3): p. 397-406.
41. Habtamu, E., Wondie, T., Aweke, S., et al., *Trachoma and Relative Poverty: A Case-Control Study.* PLoS neglected tropical diseases, 2015. **9**(11): p. e0004228-e0004228.
42. Green, M.J. and Popham, F., *Interpreting mutual adjustment for multiple indicators of socioeconomic position without committing mutual adjustment fallacies.* BMC Public Health, 2019. **19**(1): p. 10.
43. Ramke, J., Kyari, F., Mwangi, N., et al., *Cataract Services are Leaving Widows Behind: Examples from National Cross-Sectional Surveys in Nigeria and Sri Lanka.* International Journal of Environmental Research and Public Health, 2019. **16**(20): p. 3854.
44. Exeter, D.J., Zhao, J., Crengle, S., Lee, A., and Browne, M., *The New Zealand Indices of Multiple Deprivation (IMD): A new suite of indicators for social and health research in Aotearoa, New Zealand.* PLOS ONE, 2017. **12**(8): p. e0181260.
45. Gambia Bureau of Statistics (GBoS) and ICF. *The Gambia Demographic and Health Survey 2019-20: Chapter 2 Housing characteristics and household population*. 2021 [cited 2021 11.10]; Available from: https://dhsprogram.com/pubs/pdf/FR369/FR369.pdf.
46. Tarasenko, Y.N. and Schoenberg, N.E., *Self-perceived Income sufficiency and self-reported Income level among a health inequity population.* Journal of health care for the poor and underserved, 2017. **28**(2): p. 812-828.

For peer review only

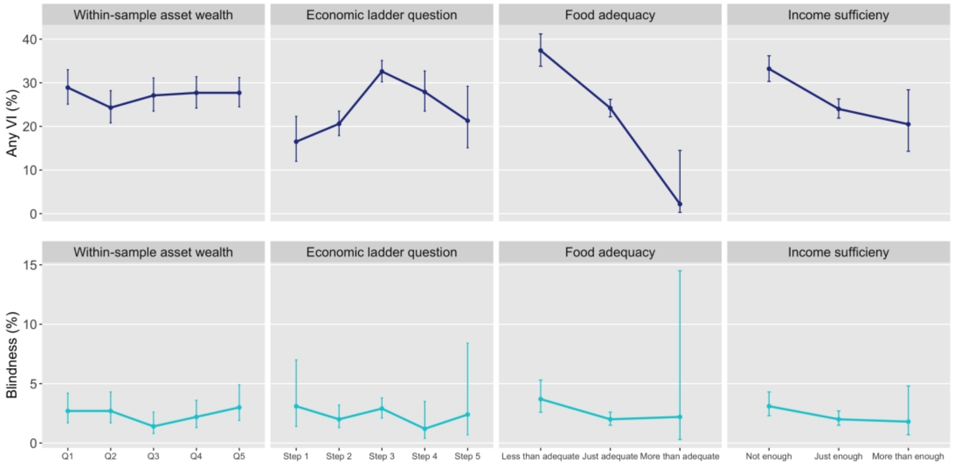

Fig 1. Age-sex weighted prevalence of blindness and any vision impairment in the population 50 years and older by four socioeconomic position variables, The Gambia 2019

1458x749mm (72 x 72 DPI)

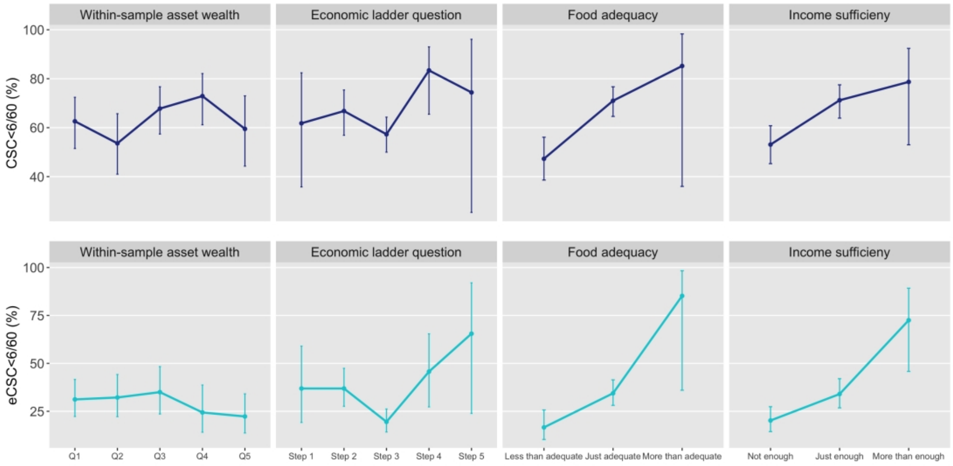

Fig 2. Age-sex weighted cataract surgical coverage and effective cataract surgical coverage (cataract surgical threshold <6/60) in the population 50 years and older by four socioeconomic position variables, The Gambia 2019

1458x749mm (72 x 72 DPI)

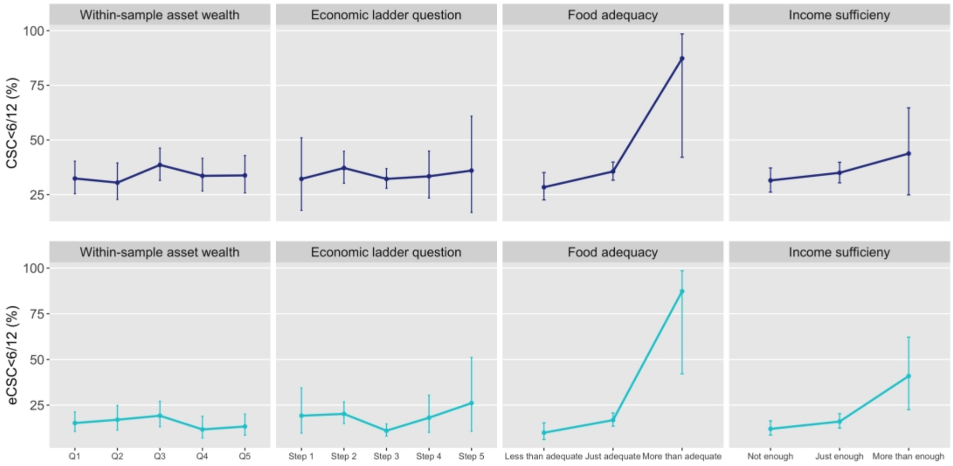

Fig 3. Age-sex weighted cataract surgical coverage and effective cataract surgical coverage (cataract surgical threshold <6/12) in the population 50 years and older by four socioeconomic position variables, The Gambia 2019

1458x749mm (72 x 72 DPI)

Supplementary Material

Table of Contents

1. EquityTool questionnaire for The Gambia 1
2. Tables of results used in Figures 1-3 2

For peer review only

1. EquityTool questionnaire for The Gambia

Released 17 Dec 2018.

Asset list

Does your household have a... (Y/N)

1. Sofa
2. Wardrobe
3. Television
4. Refrigerator
5. DVD/CD Player
6. Satellite Dish
7. Bicycle (belonging to any member of the household)

Household Characteristics

What is the main source of drinking water for members of your household? Specify if other

1. Public tap/ Standpipe
2. Piped to yard/ plot
3. Other water source

What kind of toilet facility do members of your household usually use? Specify if other

1. Flush to septic tank
2. Pit latrine without slab/ Open pit
3. Other toilet type

What is the main material of the floor? Specify if other

1. Ceramic Tile
2. Other floor material

What is the main material of the exterior walls? Specify if other

1. Cement
2. Other

Livestock

How many of the following animals does this household own...

1. Cattle
2. Milk cows or bulls
3. Horses, donkeys or mules
4. Goats
5. Sheep

For peer review only

2. Tables of results used in Figures 1-3

Age-sex weighted prevalence of any vision impairment <6/12 and blindness, and cataract surgical coverage and effective cataract surgical coverage in the population 50 years and older disaggregated by four SEP measures.

Table S1. Weighted prevalence of any vision impairment and blindness and CSC/eCSC across the 2013 DHS EquityTool national scoring within the Gambia 2019 sample aged 50 years and older

	**Quintile 1**	**Quintile 2**	**Quintile 3**	**Quintile 4**	**Quintile 5**
**Population 50+**	N=814	N=794	N=804	N=804	N=804
**Any VI (%)**	28.9 (25.1-33.0)	24.3 (20.8-28.2)	27.1 (23.5-31.1)	27.7 (24.2-31.4)	27.7 (24.5-31.2)
**Blindness (%)**	2.7 (1.7-4.2)	2.7 (1.7-4.3)	1.4 (0.8-2.6)	2.2 (1.3-3.6)	3.0 (1.9-4.9)
**Cataract surgical threshold <6/60**					
**CSC (%)**	62.6 (51.5-72.4)	53.6 (41.0-65.7)	67.8 (57.4-76.7)	72.9 (61.2-82.1)	59.5 (44.3-73.0)
**eCSC (%)**	31.2 (22.3-41.6)	32.2 (22.2-44.2)	35.0 (23.6-48.3)	24.4 (14.1-38.7)	22.3 (13.7-34.1)
**Cataract surgical threshold <6/12**					
**CSC (%)**	32.4 (25.4-40.3)	30.5 (22.8-39.5)	38.6 (31.5-46.3)	33.6 (26.7-41.6)	33.8 (25.8-42.9)
**eCSC (%)**	15.2 (10.6-21.3)	17.0 (11.3-24.8)	19.2 (13.2-27.1)	11.7 (7.0-18.9)	13.3 (8.5-20.1)

Quintiles 1=Poorest, 5=richest

Table S2. Weighted prevalence of any vision impairment and blindness and CSC/eCSC in the bottom 40% and 20% of EquityTool national scoring within the Gambia 2019 sample aged 50 years and older

		**Within-sample ET scoring**			
	**All**	**Top 60%**	**Bottom 40%**	**Top 80%**	**Bottom 20%**
**Population 50+**	N=4020	N=2412	N=1608	N=3206	N=814
**Any VI (%)**	27.2 (25.4-29.0)	27.5 (25.4-29.7)	26.7 (23.8-29.7)	26.7 (24.8-28.7)	28.9 (25.1-33.0)
**Blind (%)**	2.4 (1.9-3.0)	2.2 (1.6-3.0)	2.7 (2.0-3.7)	2.3 (1.8-3.0)	2.7 (1.7-4.2)
**Cataract surgical threshold <6/60**					
**CSC (%)**	63.1 (57.6-68.3)	66.6 (59.2-73.2)	58.4 (50.2-66.2)	63.3 (56.9-69.2)	62.6 (51.5-72.4)
**eCSC (%)**	29.1 (24.2-34.6)	27.2 (20.9-34.6)	31.7 (24.8-39.5)	28.5 (23.0-34.8)	31.2 (22.3-41.6)
**Cataract surgical threshold <6/12**					
**CSC (%)**	33.9 (30.5-37.5)	35.5 (31.1-40.1)	31.6 (26.3-37.4)	34.3 (30.4-38.4)	32.4 (25.4-40.3)
**eCSC (%)**	15.3 (12.6-18.4)	14.8 (11.5-18.9)	16.0 (12.2-20.7)	15.3 (12.3-18.9)	15.2 (10.6-21.3)

Table S3. Weighted prevalence of any vision impairment and blindness and CSC/eCSC across five self-ranked positions on an economic ladder

	**Step 1**	**Step 2**	**Step 3**	**Step 4**	**Step 5**
**Population 50+**	N=227	N=1240	N=1919	N=489	N=112
**Any VI (%)**	16.5 (12.0-22.3)	20.6 (17.9-23.5)	32.6 (30.2-35.1)	27.9 (23.5-32.7)	21.3 (15.1-29.2)
**Blindness (%)**	3.1 (1.4-7.0)	2.0 (1.3-3.2)	2.9 (2.1-3.8)	1.2 (0.4-3.5)	2.4 (0.7-8.4)
**Cataract surgical threshold <6/60**					
**CSC (%)**	61.8 (35.8-82.4)	66.8 (56.9-75.4)	57.3 (50.0-64.3)	83.4 (65.5-93.0)	74.4 (25.4-96.1)
**eCSC (%)**	36.9 (19.2-59.0)	36.9 (27.6-47.4)	19.5 (14.2-26.2)	45.7 (27.3-65.4)	65.5 (23.9-92.0)

For peer review only - http://bmjopen.bmj.com/site/about/guidelines.xhtml

**Cataract surgical threshold <6/12**					
**CSC (%)**	32.2 (17.8-51.0)	37.2 (30.2-44.8)	32.2 (27.9-36.9)	33.4 (23.5-44.9)	36.0 (16.9-60.9)
**eCSC (%)**	19.2 (9.7-34.4)	20.2 (14.9-26.8)	11.0 (8.1-14.7)	18.1 (10.1-30.4)	26.1 (10.7-51.1)

Step 1=Poorest, 5=richest

Table S4. Weighted prevalence of any vision impairment and blindness and CSC/eCSC across three subjective food adequacy levels

	**All**		
	**Less than adequate**	**Just adequate**	**More than adequate**
**Population 50+**	N=976	N=3,006	N=37
**Any VI (%)**	37.4 (33.8-41.2)	24.2 (22.2-26.2)	2.2 (0.3-14.5)
**Blindness (%)**	3.7 (2.6-5.3)	2.0 (1.5-2.6)	2.2 (0.3-14.5)
**Cataract surgical threshold <6/60**			
**CSC (%)**	47.3 (38.6-56.1)	71.0 (64.6-76.7)	85.2 (36.0-98.3)
**eCSC (%)**	16.6 (10.3-25.7)	34.4 (28.1-41.4)	85.2 (36.0-98.3)
**Cataract surgical threshold <6/12**			
**CSC (%)**	28.4 (22.6-35.1)	35.6 (31.6-39.9)	87.3 (42.1-98.5)
**eCSC (%)**	9.9 (6.2-15.3)	16.8 (13.5-20.7)	87.3 (42.1-98.5)

Table S5. Weighted prevalence of any vision impairment and blindness and CSC/eCSC across three subjective income sufficiency levels

	**All**		
	**Less than adequate**	**Just adequate**	**More than adequate**
**Population 50+**	N=1,430	N=2,418	N=172
**Any VI (%)**	33.2 (30.3-36.2)	24.0 (21.9-26.3)	20.5 (14.3-28.4)
**Blindness (%)**	3.1 (2.3-4.3)	2.0 (1.5-2.7)	1.8 (0.7-4.8)
**Cataract surgical threshold <6/60**			
**CSC (%)**	53.1 (45.3-60.8)	71.2 (63.9-77.5)	78.7 (53.0-92.4)
**eCSC (%)**	20.2 (14.4-27.4)	34.0 (26.8-42.0)	72.5 (45.8-89.2)
**Cataract surgical threshold <6/12**			
**CSC (%)**	31.5 (26.2-37.2)	35.0 (30.4-39.8)	43.8 (24.9-64.7)
**eCSC (%)**	12.0 (8.6-16.4)	16.0 (12.4-20.3)	40.9 (22.5-62.2)

For peer review only - http://bmjopen.bmj.com/site/about/guidelines.xhtml

STROBE Statement—Checklist of items that should be included in reports of ***cross-sectional studies***

	Item No	Recommendation	Page No
**Title and abstract**	1	(*a*) Indicate the study's design with a commonly used term in the title or the abstract	1-2
		(*b*) Provide in the abstract an informative and balanced summary of what was done and what was found	2-3
**Introduction**			
Background/rationale	2	Explain the scientific background and rationale for the investigation being reported	4-5
Objectives	3	State specific objectives, including any prespecified hypotheses	5
**Methods**			
Study design	4	Present key elements of study design early in the paper	5
Setting	5	Describe the setting, locations, and relevant dates, including periods of recruitment, exposure, follow-up, and data collection	5-6
Participants	6	(*a*) Give the eligibility criteria, and the sources and methods of selection of participants	5-6
Variables	7	Clearly define all outcomes, exposures, predictors, potential confounders, and effect modifiers. Give diagnostic criteria, if applicable	6-8
Data sources/ measurement	8*	For each variable of interest, give sources of data and details of methods of assessment (measurement). Describe comparability of assessment methods if there is more than one group	6-8
Bias	9	Describe any efforts to address potential sources of bias	9
Study size	10	Explain how the study size was arrived at	5
Quantitative variables	11	Explain how quantitative variables were handled in the analyses. If applicable, describe which groupings were chosen and why	8-9
Statistical methods	12	(*a*) Describe all statistical methods, including those used to control for confounding	8-9
		(*b*) Describe any methods used to examine subgroups and interactions	NA
		(*c*) Explain how missing data were addressed	9
		(*d*) If applicable, describe analytical methods taking account of sampling strategy	9
		(*e*) Describe any sensitivity analyses	NA
**Results**			
Participants	13*	(a) Report numbers of individuals at each stage of study—eg numbers potentially eligible, examined for eligibility, confirmed eligible, included in the study, completing follow-up, and analysed	9
		(b) Give reasons for non-participation at each stage	NA
		(c) Consider use of a flow diagram	NA
Descriptive data	14*	(a) Give characteristics of study participants (eg demographic, clinical, social) and information on exposures and potential confounders	10-11
		(b) Indicate number of participants with missing data for each variable of interest	10-11
Outcome data	15*	Report numbers of outcome events or summary measures	12-14

For peer review only

Main results	16	(*a*) Give unadjusted estimates and, if applicable, confounder-adjusted estimates and their precision (eg, 95% confidence interval). Make clear which confounders were adjusted for and why they were included	12-14
		(*b*) Report category boundaries when continuous variables were categorized	NA
		(*c*) If relevant, consider translating estimates of relative risk into absolute risk for a meaningful time period	NA
Other analyses	17	Report other analyses done—eg analyses of subgroups and interactions, and sensitivity analyses	NA
**Discussion**			
Key results	18	Summarise key results with reference to study objectives	15-18
Limitations	19	Discuss limitations of the study, taking into account sources of potential bias or imprecision. Discuss both direction and magnitude of any potential bias	19-20
Interpretation	20	Give a cautious overall interpretation of results considering objectives, limitations, multiplicity of analyses, results from similar studies, and other relevant evidence	20
Generalisability	21	Discuss the generalisability (external validity) of the study results	20
**Other information**			
Funding	22	Give the source of funding and the role of the funders for the present study and, if applicable, for the original study on which the present article is based	20

*Give information separately for exposed and unexposed groups.

Note: An Explanation and Elaboration article discusses each checklist item and gives methodological background and published examples of transparent reporting. The STROBE checklist is best used in conjunction with this article (freely available on the Web sites of PLoS Medicine at http://www.plosmedicine.org/, Annals of Internal Medicine at http://www.annals.org/, and Epidemiology at http://www.epidem.com/). Information on the STROBE Initiative is available at www.strobe-statement.org.

For peer review only
